# Fine-Tuning and Benchmarking Transformer Models for Multiclass Classification of Clinical Research Papers: Retrospective Modeling Study

**DOI:** 10.2196/77311

**Published:** 2026-04-29

**Authors:** Fangwen Zhou, Cynthia Lokker, Rick Parrish, R Brian Haynes, Alfonso Iorio, Ashirbani Saha, Muhammad Afzal

**Affiliations:** 1 Department of Health Research Methods, Evidence, and Impact Faculty of Health Sciences McMaster University Hamilton, ON Canada; 2 Department of Medicine Faculty of Health Sciences McMaster University Hamilton, ON Canada; 3 Department of Oncology Faculty of Health Sciences McMaster University Hamilton, ON Canada; 4 Faculty of Computing, Engineering and the Built Environment Birmingham City University Birmingham United Kingdom

**Keywords:** classification, deep learning, information science, medical informatics, natural language processing

## Abstract

**Background:**

The exponential growth of digital information has led to an unprecedented expansion in the volume of unstructured text data. Efficient classification of these data is critical for timely evidence synthesis and informed decision-making in health care. Machine learning techniques have shown considerable promise for text classification tasks. However, multiclass classification of papers by study publication type has been largely overlooked compared to binary or multilabel classification. Addressing this gap could significantly enhance knowledge translation workflows and support systematic review processes.

**Objective:**

This study aimed to fine-tune and evaluate domain-specific transformer-based language models on a gold-standard dataset for multiclass classification of clinical literature into mutually exclusive categories: original studies, reviews, evidence-based guidelines, and nonexperimental studies.

**Methods:**

The titles and abstracts of McMaster’s Premium Literature Service (PLUS) dataset comprising 162,380 papers were used for fine-tuning seven domain-specific transformers. Clinical experts classified the papers into four mutually exclusive publication types. PLUS data were split in an 80:10:10 ratio into training, validation, and testing sets, with the Clinical Hedges dataset used for external validation. A grid search evaluated the impact of class weight (CW) adjustments, learning rate (LR), batch size (BS), warmup ratio, and weight decay (WD), totaling 1890 configurations. Models were assessed using 10 metrics, including the area under the receiver operating characteristic curve (AUROC), the *F*_1_-score (harmonic mean of precision and recall), and Matthew’s correlation coefficient (MCC). The performance of individual classes was assessed using a one-to-rest approach, and overall performance was assessed using the macro average. Optimal models identified from validation results were further tested on both PLUS and Clinical Hedges, with calibration assessed visually.

**Results:**

Ten best-performing models achieved macro AUROC≥0.99, *F*_1_-score≥0.89, and MCC≥0.88 on the validation and testing sets. Performance declined on Clinical Hedges. Models were consistently better at classifying original studies and reviews. Biomedical Bidirectional Encoder Representations from Transformers (fine-tuned on biomedical text; BioBERT)–based models had superior calibration performance, especially for original studies and reviews. Optimal configurations for search included lower LRs (1 × 10–5 and 3 × 10–5), midrange BSs (32–128), and lower WD (0.005-0.010). CW adjustments improved recall but generally reduced performance on other metrics. Models generally struggled with accurately classifying nonexperimental and guideline studies, potentially due to class imbalance and content heterogeneity.

**Conclusions:**

This study used a comprehensive hyperparameter search to highlight the effectiveness of fine-tuned transformer models, notably BioBERT variants, for multiclass clinical literature classification. Although class weighting generally decreased overall performance, addressing class imbalance through alternative methods, such as hierarchical classification or targeted resampling, warrants future exploration. Hyperparameter configurations were crucial for robust performance, aligning with the previous literature. These findings support future modeling research and practical deployment in human-in-the-loop systems to support knowledge synthesis and translation workflows with the findings from this work.

## Introduction

### Background

The exponential growth of health evidence has led to an unprecedented expansion in the volume of unstructured text data. For instance, PubMed, a leading repository of biomedical literature, has over 36 million papers indexed as of 2025, with approximately 1 million new papers added annually [1,2]. This highlights the critical need for automated methods to classify, organize, and retrieve relevant information efficiently.

### Machine Learning for Text Classification Tasks

Text classification is a key natural language processing (NLP) task that involves assigning predefined categories to unstructured text [3,4]. It underpins various applications, including sentiment analysis and spam detection [5,6]. Deep learning, compared to rule-based [7-10] or shallow [8,11-13] learning approaches, has significantly advanced text classification by automating feature extraction and improving contextual understanding [14]. Recurrent neural networks (RNNs), particularly their bidirectional and gated variants, demonstrate better performance by capturing sequential relationships in text [15-17]. However, they suffer from computational inefficiencies, vanishing gradients, and limitations in processing long-range dependencies [18-21].

Transformer-based models, such as Bidirectional Encoder Representations from Transformers (BERT), have overcome these challenges by introducing self-attention mechanisms that consider all tokens in parallel, improving both efficiency and contextual embedding [22-25]. Pretrained models, including domain-specific variants such as Biomedical Bidirectional Encoder Representations from Transformers (fine-tuned on biomedical text; BioBERT) [26], Scientific Bidirectional Encoder Representations from Transformers (SciBERT) [27], and Biomedical Document Link Bidirectional Encoder Representations from Transformers (BioLinkBERT) [28], leverage large-scale biomedical corpora to enhance classification performance in specialized fields. Transfer learning further optimizes these models by adapting them to new tasks with minimal labeled data, making them highly effective for text classification [29].

### Medical Literature Classification

Traditional indexing methods, such as Medical Subject Headings (MeSH), improve literature retrieval but suffer from delays (often taking months for new papers to be indexed) and inconsistencies due to subjective keyword selection [30,31]. Machine learning (ML)–based approaches have been explored to classify medical literature into binary, multilabel, and multiclass categories. Early rule-based systems, such as the Medical Text Indexer (MTI), automated MeSH indexing, but newer neural network models, such as MTI-NeXt, significantly improved recall and efficiency [32,33]. ML has also been used to classify papers based on study type, topic, or methodological rigor, supporting systematic reviews and clinical decision-making [34-38].

Binary classifiers have been used to assess methodological soundness or relevance to systematic reviews [39-51]. However, multiclass classification, where papers must be assigned a single category among multiple mutually exclusive classes, presents additional challenges [52-55]. Traditional approaches decompose the problem into multiple binary tasks using one-versus-one (OvO) or one-versus-rest (OvR) methods, but these are computationally expensive or, for OvR, yield ambiguous or poorly calibrated predictions when multiple classifiers fire [52-55]. In contrast, transformers natively support multiclass classification through multiheaded SoftMax activation [56]. Yet, limited studies have examined their effectiveness in the multiclass classification of clinical literature, and existing work has not fully addressed the impact of pretraining, class imbalance, or optimal hyperparameter selection [57,58].

### Objective

The objective of this research was to fine-tune and evaluate the performance of seven domain-specific, encoder-only transformers using various hyperparameter configurations on the task of multiclass classification of published clinical literature into original studies, reviews, evidence-based guidelines, and nonexperimental studies. We leveraged two datasets curated and annotated by the Health Information Research Unit (HIRU) at McMaster University, with the goal of identifying a multiclass model to support evidence processing early after publication and before indexing tasks are completed.

## Methods

### Data Source and Preprocessing

HIRU is a pioneer in providing curated evidence services targeted to clinicians worldwide. Originally, HIRU curated the Clinical Hedges dataset in 2000, which comprises classifications and critical appraisal of ~49,000 papers across 161 journals indexed in MEDLINE [59]. Each paper was manually classified by domain experts into mutually exclusive study formats, and clinically relevant papers that reported the findings of an original study or a review were subsequently labeled for research purposes and assessed for methodological rigor.

Subsequently, McMaster’s Premium Literature Service (PLUS) dataset, which classifies and appraises clinical research reports indexed in PubMed, was initiated in 2003. Through the ongoing PLUS process, HIRU has continued to grow a database of clinically relevant papers appraised at the time of publication using Clinical Hedges’s criteria and classifications with some modifications. Specifically, PLUS involves automated daily searches of PubMed using a sensitive methods filter adapted from Clinical Queries and applied to ~125 journal titles (with some expansion during the COVID-19 pandemic to all journals and using a COVID-19–based filter) [60]. To date, the PLUS dataset includes over 150,000 clinical papers classified by publication type into mutually exclusive classes: (1) original studies, (2) reviews, (3) evidence-based guidelines, or (4) nonexperimental studies (for indexed items such as case studies, general or philosophical discussions of a topic without original observations and without a statement that the purpose was to review or appraise a body of knowledge, secondary publications, letters, or commentaries) [61].

All papers that were retrieved and assessed through PLUS from inception to 2023 were included. The dataset was randomly split in an 80:10:10 ratio into training, validation, and testing sets, and these subsets were termed PLUS-train, PLUS-validate, and PLUS-test, respectively. The original Clinical Hedges dataset was used for external testing. The titles and abstracts of papers were combined and tokenized using the pretrained model’s corresponding tokenizer and were used as inputs. Inputs longer than the maximum token length of 512 were truncated, and shorter inputs were padded to ensure uniform input length. No text normalization was performed.

### Model Configurations and Hyperparameters

We selected seven pretrained models to fine-tune, based on their performance in the previous literature and the Biomedical Language Understanding and Reasoning Benchmark (BLURB) leaderboard [26-28,62-68]. Each model was trained with or without class weight (CW) adjustments. The training process minimized cross-entropy loss. We used a linear learning rate (LR) scheduler. The AdamW optimizer [69] was used with the default β1 of 0.9 and β2 of 0.999. We enabled mixed precision training for faster training and less memory usage. In the case of insufficient memory for a particular batch size (BS), we enabled gradient accumulation. We fine-tuned for at most 10 epochs with an early stopping patience of 3, where training was prematurely terminated if the cross-entropy loss on the PLUS-validate set failed to improve for 3 consecutive epochs. The weights from the epoch with the lowest loss on the PLUS-validate set were selected to mitigate overfitting. A grid search of the LR, BS, warmup ratio (WR), and weight decay (WD) was conducted. Overall, models were trained with 1890 configurations (Table 1). Each model’s output layer produced four logits, one for each class, which were converted to probabilities using the SoftMax function. During fine-tuning, the models minimized categorical cross-entropy loss.

**Table 1:** Model configuration grid.

Parameter	Count	Values
Pretrained model	7	BioBERT^a^ [26], BioELECTRA^b^ [67], BioLinkBERT^c^ [28], BiomedBERT^d^ (abstracts only) [70], BiomedBERT (abstracts+full text) [70], SciBERT^e^-cased [27], SciBERT-uncased [27]
CW^f^ adjustment	2	Yes, no
LR^g^	3	1 × 10^–5^, 3 × 10^–5^, 5 × 10^–5^
BS^h^	5	16, 32, 64, 128, 256
WR^i^	3	0.05, 0.10, 0.20
WD^j^	3	0.005, 0.010, 0.015

^a^BioBERT: Biomedical Bidirectional Encoder Representations from Transformers (fine-tuned on biomedical text).

^b^BioELECTRA: Biomedical Efficiently Learning an Encoder that Classifies Token Replacements Accurately.

^c^BioLinkBERT: Biomedical Document Link Bidirectional Encoder Representations from Transformers.

^d^BiomedBERT: Biomedical Bidirectional Encoder Representations from Transformers (trained entirely on biomedical text). Formerly known as PubMedBERT.

^e^SciBERT: Scientific Bidirectional Encoder Representations from Transformers.

^f^CW: class weight.

^g^LR: learning rate.

^h^BS: batch size.

^i^WR: warmup ratio.

^j^WD: weight decay.

#### Class Weight Calculation

CWs were calculated using the following formula:



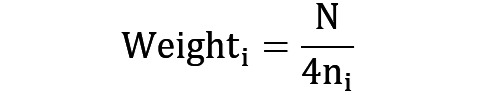



where N is the total number of samples and n_i_ is the number of samples in class “i.”

### Model Evaluation

#### Macrolevel Performance of Models

We presented model results using the macro average of cross-entropy loss, the Brier score, the area under the receiver operating characteristic curve (AUROC), the average precision (AP), recall, precision, accuracy, *F*_1_-scores (harmonic mean of precision and recall) and *F*_2_-scores (harmonic mean of precision and recall, with an emphasis on recall), and Matthew’s correlation coefficient (MCC). The interpretation of these metrics is tabulated in Table S1 in Multimedia Appendix 1. The macro average was selected to mitigate bias toward more prevalent classes.

We grouped models based on the values of the six configurations in Table 1 to assess how model performance was affected. Performance differences on the validation set between model configurations were presented using means and 95% CIs and visualized using bar plots.

#### Selecting and Testing the Best Models

We narrowed the models to those that achieved the best macro average performance on one or more evaluation metrics on the validation set. These models were then further evaluated on the testing set and Clinical Hedges [71]. For individual classes, we used an OvR approach, where each class was treated as a separate binary classification problem. Specifically, for each class, we labeled instances of that class as the positive class and instances of all other classes as the negative class. Metrics on evidence-based guidelines were not considered for the Clinical Hedges test as the dataset did not contain this class. We used AUROC, the *F*_1_-score, and the MCC as the primary evaluation metrics. We estimated 95% CIs using bootstrapping (ie, repeated sampling with replacement) over 1000 iterations [72]. Confusion matrices were provided to examine classification errors between classes. Calibration plots of each model were presented and visually inspected. Points below the diagonal indicated that the true proportion of the class is lower than the average predicted probability, meaning the model is overconfident for that class. Points above the diagonal indicated underconfidence. A perfect diagonal line meant the model is perfectly calibrated, reflecting reliable performance across the full probability range.

### Hardware and Software

All fine-tuning was performed on the Cedar cluster provided by the Digital Research Alliance of Canada. Each model was trained on a single NVIDIA V100 Volta graphics processing unit (GPU; 32 GB memory), with access to eight central processing unit cores and 40 GB of memory. Details of the software environment are listed in Table S2 in Multimedia Appendix 1. All software development was carried out using Visual Studio Code and Python 3.11.5. Pretrained models were obtained using the Hugging Face Transformers library, while evaluation tasks were performed with PyTorch. Data management and statistical analysis were conducted with Pandas, NumPy, and scikit-learn, and matplotlib was used for data visualization.

### Ethical Considerations

This study involved the processing of only published biomedical and clinical literature, so ethics approval was not required.

## Results

### Characteristics of Datasets

The training of all 1890 models took 5162.42 GPU-hours. Each model used an average of 2.73 (SD 0.52) hours to train. A total of 162,380 records from PLUS were used in this study, of which 129,904 (80%) were used for training (Table 2). The class distribution of the training, validation, and testing sets was similar, in which original studies, reviews, evidence-based guidelines, and nonexperimental studies accounted for approximately 65.0% (n=84,398), 24.4% (n=31,684), 1.8% (n=2318), and 8.9% (n=11,504) of the dataset, respectively. Clinical Hedges included a total of 48,044 papers, of which most were original (n=25,747, 53.6%) or nonexperimental (n=19,234, 40.0%) studies. There were no evidence-based guidelines in Clinical Hedges.

**Table 2:** Characteristics of datasets.

Dataset	Study type				Total (N=162,380), n (%)	
	Original studies, n (%)	Reviews, n (%)	Evidence-based guidelines, n (%)	Nonexperimental studies^a^, n (%)		
PLUS^b^-train	84,398 (65.0)	31,684 (24.4)	2318 (1.8)	11,504 (8.9)	129,904 (80.0)	
PLUS-validate	10,640 (65.5)	3898 (24.0)	293 (1.8)	1407 (8.7)	16,238 (10.0)	
PLUS-test	10,496 (64.6)	3989 (24.6)	318 (2.0)	1435 (8.8)	16,238 (10.0)	
PLUS total	105,534 (65.0)	39,571 (24.4)	2929 (1.8)	14,346 (8.8)	162,380 (100.0)	
Clinical Hedges	25,747 (53.6)	3063 (6.4)	N/A^c^	19,234 (40.0)	48,044 (29.6)	

^a^Case studies, general or philosophical discussions without original observations or a clear purpose to review or appraise a body of knowledge, secondary publications, letters, or commentaries.

^b^PLUS: Premium Literature Service.

^c^N/A: not applicable. The Clinical Hedges dataset includes papers from 2000; guidelines were not as prevalent at the time, and the label was not used.

### Aggregated Performance on PLUS-Validate

The metric macro averages of the different model configurations on the PLUS-validate set are summarized in Table 3, Figures S1-S6 in Multimedia Appendix 1, and Table S1 in Multimedia Appendix 2. Models without CW adjustments showed better performance across all metrics except for recall and the *F*_2_-score (Figure S2 in Multimedia Appendix 1). Models with lower LRs generally performed better than those with higher LRs (Figure S3 in Multimedia Appendix 1). A BS of 16 resulted in worse performance, and a BS of 256 had wider variance; the other sizes showed relatively mixed performance (Figure S4 in Multimedia Appendix 1). There was no clear trend among the WRs (Figure S5 in Multimedia Appendix 1), and a smaller WD marginally improved performance (Figure S6 in Multimedia Appendix 1).

**Table 3:** Macro averages of evaluation metrics on the PLUS^a^-validate set by model configuration parameters.

Configuration and models			AUROC^b^, mean (95% CI)		*F*_1_-score, mean (95% CI)	MCC^c^, mean (95% CI)
**Pretrained model**						
	BioBERT^d^	0.994 (0.994-0.994)		0.897 (0.895-0.898)		0.884 (0.883-0.886)
	BioELECTRA^e^	0.993 (0.993-0.994)		0.896 (0.895-0.897)		0.883 (0.882-0.884)
	BioLinkBERT^f^	0.994 (0.994-0.994)		0.898 (0.896-0.899)		0.885 (0.884-0.886)
	BiomedBERT^g^ (abstract only)	0.992 (0.990-0.995)		0.895 (0.891-0.899)		0.882 (0.876-0.887)
	BiomedBERT (abstract+full text)	0.994 (0.994-0.994)		0.897 (0.895-0.898)		0.884 (0.883-0.886)
	SciBERT^h^-cased	0.994 (0.994-0.994)		0.894 (0.893-0.896)		0.881 (0.880-0.883)
	SciBERT-uncased	0.994 (0.994-0.994)		0.897 (0.896-0.898)		0.884 (0.883-0.885)
**CW^i^** **adjustment**						
	No	0.995 (0.995-0.995)		0.903 (0.903-0.904)		0.891 (0.890-0.891)
	Yes	0.993 (0.992-0.993)		0.889 (0.888-0.890)		0.877 (0.875-0.878)
**LR^j^**						
	1 × 10^–5^	0.995 (0.994-0.995)		0.901 (0.900-0.901)		0.889 (0.888-0.889)
	3 × 10^–5^	0.993 (0.992-0.995)		0.895 (0.894-0.897)		0.883 (0.880-0.885)
	5 × 10^–5^	0.993 (0.993-0.993)		0.892 (0.891-0.893)		0.879 (0.878-0.880)
**BS^k^**						
	16	0.992 (0.992-0.993)		0.895 (0.895-0.896)		0.882 (0.881-0.883)
	32	0.993 (0.993-0.994)		0.896 (0.895-0.897)		0.884 (0.883-0.885)
	64	0.994 (0.994-0.994)		0.896 (0.895-0.897)		0.884 (0.882-0.885)
	128	0.995 (0.995-0.995)		0.897 (0.896-0.898)		0.885 (0.884-0.886)
	256	0.994 (0.992-0.996)		0.896 (0.893-0.899)		0.884 (0.880-0.888)
**WR^l^**						
	0.05	0.994 (0.994-0.994)		0.895 (0.895-0.896)		0.883 (0.882-0.884)
	0.10	0.994 (0.994-0.994)		0.896 (0.895-0.897)		0.883 (0.883-0.884)
	0.20	0.993 (0.992-0.995)		0.897 (0.895-0.899)		0.884 (0.882-0.887)
**WD^m^**						
	0.005	0.994 (0.994-0.994)		0.896 (0.895-0.897)		0.884 (0.883-0.885)
	0.010	0.994 (0.994-0.994)		0.896 (0.895-0.897)		0.884 (0.883-0.885)
	0.015	0.993 (0.992-0.995)		0.896 (0.894-0.898)		0.883 (0.881-0.885)

^a^PLUS: Premium Literature Service.

^b^AUROC: area under the receiver operating characteristic curve.

^c^MCC: Matthew’s correlation coefficient.

^d^BioBERT: Biomedical Bidirectional Encoder Representations from Transformers (fine-tuned on biomedical text).

^e^BioELECTRA: Biomedical Efficiently Learning an Encoder that Classifies Token Replacements Accurately.

^f^BioLinkBERT: Biomedical Document Link Bidirectional Encoder Representations from Transformers.

^g^BiomedBERT: Biomedical Bidirectional Encoder Representations from Transformers (trained entirely on biomedical text). Formerly known as PubMedBERT.

^h^SciBERT: Scientific Bidirectional Encoder Representations from Transformers.

^i^CW: class weight.

^j^LR: learning rate.

^k^BS: batch size.

^l^WR: warmup ratio.

^m^WD: weight decay.

### Best-Performing Models

No model had the best macro average in more than one metric on the validation set. The configurations of the 10 best models can be found in Table S3 in Multimedia Appendix 1. BioBERT (n=4, 40%) and BiomedBERT (n=4, 40%) were the most frequently used pretrained architectures, followed by BioLinkBERT (n=1, 10%) and SciBERT-uncased (n=1, 10%). Most models did not use class weighting (n=8, 80%), and the most common LRs were 1 × 10^–5^ (n=4, 40%) and 3 × 10^–5^ (n=4, 40%). The majority of models had a weight decay of either 0.010 (n=3, 30%) or 0.015 (n=5, 50%). For the BS, 2 (20%), 2 (20%), 2 (20%), and 4 (40%)models used a value of 16, 64, 128, and 256, respectively. For the WR, 3 (30%), 3 (30%), and 4 (40%)models used a value of 0.05, 0.10, and 0.20, respectively.

Table 4 and Table S2 in Multimedia Appendix 2 present the performance of models that achieved the best macro average in AUROC (BiomedBERT: CW=no; LR=3 × 10^–5^; BS=256; WR=0.20; WD=0.005), the *F*_1_-score (BioBERT: CW=no; LR=5 × 10^–5^; BS=64; WR=0.10; WD=0.015), and the MCC (BiomedBERT: CW=no; LR=1 × 10^–5^; BS=16; WR=0.20; WD=0.005) on the PLUS-validate set. On the PLUS subsets, the macro average AUROC, *F*_1_-score, and MCC were 0.993-0.996, 0.904-0.914, and 0.890-0.902, respectively. Using an OvR approach, the classification performance, from best to worst, was original studies, reviews, nonexperimental studies, and evidence-based guidelines. On Clinical Hedges, macro average performance was significantly lower in AUROC (0.957-0.963), the *F*_1_-score (0.817-0.826), and the MCC (0.754-0.765). Class-wise, the models were the best at classifying original studies and the worst at reviews.

**Figure 1:**
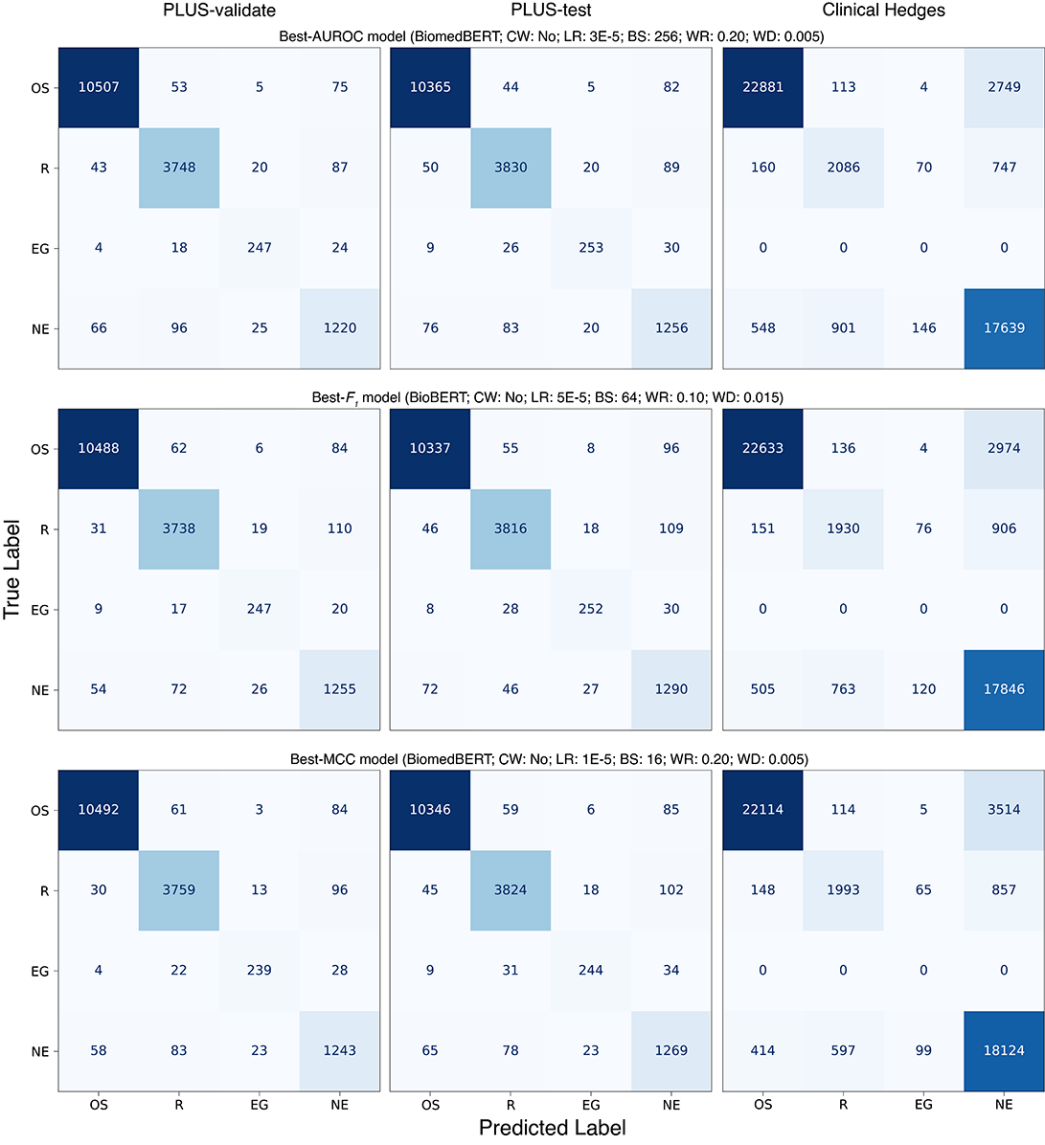
Confusion matrices of top models. AUROC: area under the receiver operating characteristic curve; BioBERT: Biomedical Bidirectional Encoder Representations from Transformers (fine-tuned on biomedical text); BiomedBERT: Biomedical Bidirectional Encoder Representations from Transformers (trained entirely on biomedical text); BS: batch size; CW: class weight; LR: learning rate; OS: original study; PLUS: Premium Literature Service; R: review; EG: evidence-based guideline; NE: nonexperimental study;. WD: weight decay; WR: warmup ratio.

**Figure 2:**
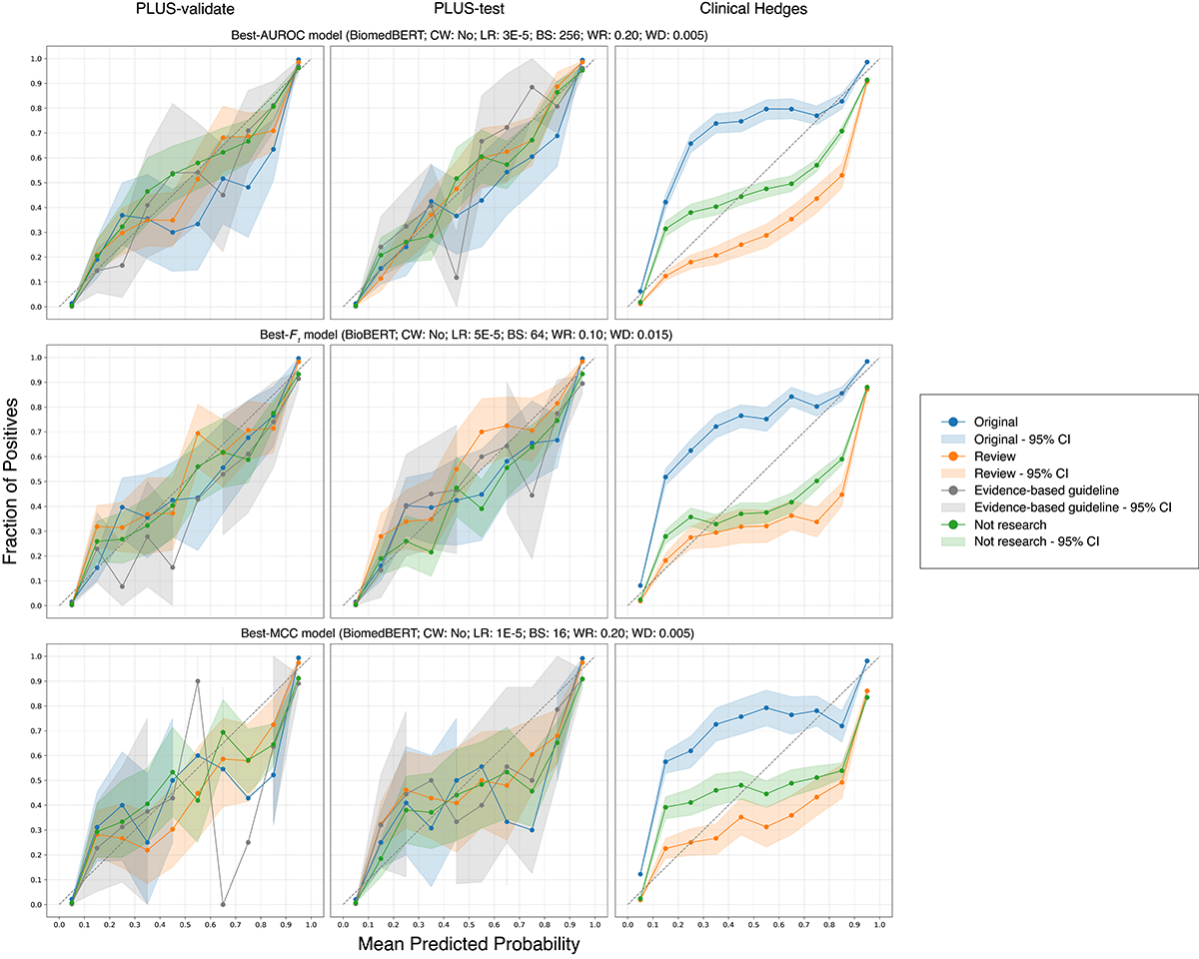
Calibration plots of top models. AUROC: area under the receiver operating characteristic curve; BioBERT: Biomedical Bidirectional Encoder Representations from Transformers (fine-tuned on biomedical text); BiomedBERT: Biomedical Bidirectional Encoder Representations from Transformers (trained entirely on biomedical text); BS: batch size; CW: class weight; LR: learning rate; PLUS: Premium Literature Service; WD: weight decay; WR: warmup ratio.

The confusion matrices (Figure 1) of the three models indicated that common confusions occurred between nonexperimental studies misclassified as reviews or original studies, and vice versa, on Clinical Hedges. No notable pattern of confusion was present on the PLUS subsets. Upon visual inspection of the calibration plots (Figure 2), the best-AUROC model (BiomedBERT: CW=no; LR=3 × 10^–5^; BS=256; WR=0.20; WD=0.005) had the best calibration among the three models. On the PLUS subsets, the models were generally well calibrated, considering that few papers were predicted with probability≥0.10 or ≤0.90, evident by the width of the 95% CI. Models demonstrated poorer calibration on Clinical Hedges, where they were underconfident on original studies and overconfident on reviews and evidence-based guidelines.

The results of all 10 best-performing models across all metrics on PLUS-validate, PLUS-test, and Clinical Hedges can be found in Tables S4, S5, and S6 in Multimedia Appendix 1, respectively. Confusion matrices and calibration plots for the other seven models can be found in Figures S7-S12 and Figures S13-S19 in Multimedia Appendix 1, respectively. In general, the best-recall model (SciBERT-uncased: CW=yes; LR=3 × 10^–5^; BS=256; WR=0.05; WD=0.010) had worse performance than others, and all other models demonstrated similar performance and trends without meaningful differences. The best-loss (BioBERT: CW=no; LR=5 × 10^–5^; BS=256; WR=0.10; WD=0.015; Figure S13 in Multimedia Appendix 1), best-Brier score (BioBERT: CW=no; LR=1 × 10^–5^; BS=64; WR=0.20; WD=0.015; Figure S14 in Multimedia Appendix 1), and best-accuracy (BioBERT: CW=no; LR=1 × 10^–5^; BS=256; WR=0.05; WD=0.015; Figure S18 in Multimedia Appendix 1) models were better calibrated than the others on the PLUS subsets. Calibration on Clinical Hedges was mixed, with all models being underconfident on original studies and overconfident in the other classes, with the best-AP model (BiomedBERT: CW=no; LR=1 × 10^–5^; BS=128; WR=0.05; WD=0.010; Figure S15 in Multimedia Appendix 1) having the best performance based on visual inspection.

**Table 4:** Performance of the top models on AUROC^a^, *F*_1_-score, and MCC^b^.

Model (best metric; CW^c^, LR^d^, BS^e^, WR^f^, WD^g^) and class		PLUS^h^-test				Clinical Hedges		
		AUROC, score (bootstrapped 95% CI)	*F*_1_-score, score (bootstrapped 95% CI)	MCC, score (bootstrapped 95% CI)	AUROC, score (bootstrapped 95% CI)		*F*_1_-score, score (bootstrapped 95% CI)	MCC, score (bootstrapped 95% CI)
**BiomedBERT^i^** **(AUROC; no, 3 × 10^–5^** **, 256, 0.20, 0.005)**								
	Original study	0.997 (0.996-0.998)	0.987 (0.986-0.989)	0.964 (0.960-0.968)	0.974 (0.973-0.976)		0.928 (0.925-0.930)	0.855 (0.850-0.860)
	Review	0.996 (0.995-0.997)	0.961 (0.957-0.965)	0.948 (0.942-0.954)	0.953 (0.949-0.957)		0.677 (0.664-0.690)	0.655 (0.641-0.669)
	Evidence-based guideline	0.994 (0.991-0.997)	0.821 (0.784-0.853)	0.818 (0.780-0.850)	N/A^j^		N/A	N/A
	Nonexperimental study	0.991 (0.988-0.993)	0.869 (0.856-0.881)	0.856 (0.841-0.869)	0.961 (0.959-0.962)		0.874 (0.871-0.878)	0.785 (0.780-0.791)
	Macro average	0.995 (0.993-0.996)	0.910 (0.899-0.919)	0.897 (0.885-0.906)	0.963 (0.961-0.964)		0.826 (0.821-0.831)	0.765 (0.759-0.771)
**BioBERT^k^ (*F*_1_-score;** ** no, 5 × 10^–5^** **, 64, 0.10, 0.015)**								
	Original study	0.997 (0.996-0.998)	0.986 (0.985-0.988)	0.962 (0.957-0.966)	0.972 (0.970-0.973)		0.923 (0.921-0.926)	0.848 (0.843-0.852)
	Review	0.996 (0.995-0.997)	0.962 (0.958-0.966)	0.950 (0.944-0.955)	0.948 (0.944-0.952)		0.655 (0.642-0.669)	0.633 (0.620-0.648)
	Evidence-based guideline	0.994 (0.990-0.997)	0.809 (0.775-0.839)	0.805 (0.771-0.836)	N/A		N/A	N/A
	Nonexperimental study	0.992 (0.989-0.993)	0.872 (0.859-0.883)	0.859 (0.846-0.872)	0.958 (0.956-0.960)		0.871 (0.868-0.875)	0.781 (0.775-0.786)
	Macro average	0.995 (0.993-0.996)	0.907 (0.897-0.917)	0.894 (0.883-0.904)	0.959 (0.957-0.961)		0.817 (0.812-0.822)	0.754 (0.748-0.760)
**BiomedBERT (MCC; no, 1 × 10^–5^** **, 16, 0.20, 0.005)**								
	Original study	0.996 (0.995-0.997)	0.987 (0.986-0.989)	0.964 (0.959-0.968)	0.967 (0.965-0.968)		0.913 (0.911-0.916)	0.833 (0.828-0.838)
	Review	0.996 (0.995-0.997)	0.958 (0.954-0.963)	0.945 (0.939-0.951)	0.949 (0.945-0.953)		0.691 (0.677-0.704)	0.673 (0.658-0.687)
	Evidence-based guideline	0.992 (0.987-0.996)	0.801 (0.763-0.836)	0.798 (0.761-0.833)	N/A		N/A	N/A
	Nonexperimental study	0.989 (0.986-0.992)	0.868 (0.854-0.880)	0.855 (0.840-0.868)	0.954 (0.953-0.956)		0.869 (0.865-0.872)	0.776 (0.771-0.781)
	Macro average	0.993 (0.992-0.995)	0.904 (0.893-0.914)	0.890 (0.879-0.902)	0.957 (0.955-0.959)		0.824 (0.819-0.830)	0.761 (0.754-0.767)

^a^AUROC: area under the receiver operating characteristic curve.

^b^MCC: Matthew’s correlation coefficient.

^c^CW: class weight.

^d^LR: learning rate.

^e^BS: batch size.

^f^WR: warmup ratio.

^g^WD: weight decay.

^h^PLUS: Premium Literature Service.

^i^BiomedBERT: Biomedical Bidirectional Encoder Representations from Transformers (trained entirely on biomedical text). Formerly known as PubMedBERT.

^j^N/A: not applicable.

^k^BioBERT: Biomedical Bidirectional Encoder Representations from Transformers (fine-tuned on biomedical text).

## Discussion

### Principal Findings

For this study, we leveraged an expert-annotated dataset of over 150,000 papers for tuning state-of-the-art pretrained encoder transformer models using a comprehensive grid search and evaluation process. The findings highlight the effects of hyperparameter settings in text-based multiclass problems. The findings for the case of classifying papers by publication type can inform future research on multiclass classification of clinical text and expected performance when the models are deployed in digital systems to support curating evidence-based clinical literature.

#### Model Performance

We identified 10 top-performing models and tested these in a set and Clinical Hedges data. These models demonstrated high performance on all study classes with macro AUROC≥0.99, *F*_1_-score≥0.89, and MCC≥0.88 on both validation and testing sets. BioBERT-based models tended to have better calibration. These models have the potential to reliably add annotations in the PLUS workflow, especially for original studies and reviews. However, the performance on nonexperimental studies and evidence-based guideline reports was noticeably worse, presumably due to class imbalance and the intrinsic heterogeneity present in these classes of papers [61]. The performance on Clinical Hedges was also worse, and this could be a result of differences in classification criteria and data drift over time since 2000, as well as changes in the reporting structure of papers [73-75].

For PLUS, the calibration plots show that most papers are predicted with high confidence (≤10% or ≥90%), and these predictions generally align well with true proportions, indicating reliable classification performance. For Clinical Hedges, the calibration curve for the original class lies mostly above the diagonal, showing that the true proportion of original papers is higher than the predicted probabilities. This suggests the models tend to underestimate their prevalence in this dataset and highlights the importance of monitoring the calibration performance to use the models’ predictions responsibly.

#### Effect of Model Configurations

All seven pretrained models have the potential to achieve a satisfactory performance for classification in this context when the ideal hyperparameters are combined. However, among the top 10 models, the calibration of the BioBERT models was generally better. For this reason, we believe that BioBERT has the best potential for future examination and deployment for this task.

Typically, the class imbalance should be addressed through resampling or CW adjustments [76,77]. We found that CW adjustments generally resulted in worse macro average performance, except for recall and those metrics that heavily prioritize recall. Eight of the ten best-performing models did not include CW adjustments, and no notable degradation in performance was seen in evidence-based guidelines and nonexperimental studies compared to the two models that included CW adjustments. Intuitively, with substantial class imbalance, CW adjustment encourages more positive predictions for those classes, increasing both true positives and false positives. Due to drastically lower prevalence of the minority classes, the latter dominated, resulting in a decline in summary metrics despite an increase in recall. This is consistent with the prior literature that found that class imbalance correction techniques may not improve classification performance [78-80]. Therefore, unless recall for the minority classes is a priority, omitting CW adjustments was preferable in our experiments. Nevertheless, it is apparent that class balance remains an issue after adjustments. Interestingly, in our previous binary classification experiments using Light Gradient Boosting Machine (LightGBM) [41] and BERT models [42], resampling typically resulted in better performance across most metrics.

The LR is one of the most important hyperparameters in fine-tuning encoder transformers [57,81]. Our experiment suggests that a lower LR results in better, more consistent performance. This is consistent with the previous literature [81]. which suggests that higher LRs may not be suitable for finetuning in this context. We recommend that future research on similar tasks include experiments with an LR of 3 × 10^–5^ or lower for BERT and Efficiently Learning an Encoder that Classifies Token Replacements Accurately (ELECTRA) models. A BS of 256 was the largest we experimented with. We found that a BS of 16 performs much worse than others, and a BS of 256 resulted in more inconsistent performance, as evident through the wider 95% CI. Although 2 of the 10 best-performing models used a BS of 16, their performance was not significantly better than the others. For computational resource efficiency, future experiments should search among BSs of 32 or greater [82-84].

Regarding the WR, we could not identify any meaningful patterns. A larger ratio seems to improve precision and worsen recall, but the effects are marginal, with a <1% difference on average. Performance on other metrics seems to be mixed as well. Future studies may wish to further explore the effects of the WR in depth. For the WD, a value of 0.005 or 0.010 offered similar performance, with 0.005 being slightly better. A WD of 0.015 should be avoided as the performance was worse and inconsistent.

#### Practice Implications

Since August 2022, HIRU has implemented a binary BioBERT classifier with the probability threshold set at 99% sensitivity to confidently exclude irrelevant papers for PLUS [42]. Results indicate that there is a work saving of >60%, and this indicates the strong potential for these models to improve efficiency. This multiclass experiment serves as the next step in the automation of the costly manual appraisal process. For PLUS, the best-performing models would be satisfactory for deployment in human-in-the-loop systems to aid in paper type classification, recognizing this in one step in a series for the human experts. Based on calibration performance and prior implementation experience, a promising strategy would be to automatically classify papers assigned a high probability threshold, such as ≥95% for a given class, while treating lower-confidence predictions as unsure and routing for manual annotation. Providing human reviewers with probability scores can also support their decision-making when classifying papers. New annotations for additional training may also alleviate the performance decrease over time due to data drift [75,85-87].

Regarding other services, Health Evidence, a database focused on appraising and disseminating high-quality public health knowledge synthesis evidence [88], has introduced ML into its workflow in a similar fashion [89]. Specifically, the DistillerSR AI Preview & Rank feature was used, and the minimum probability threshold that would result in perfect recall was identified. Subsequently, the threshold was increased until five false negatives occurred, resulting in doubling of the specificity from 36% to 72%. Over a 3-year implementation period, there was a 70% reduction in the manual screening effort, with an estimated time saving of 382 person-hours. These findings echo the success that HIRU has had and the potential for further efficiency gains from the implementation of the multiclass models for HIRU.

Externally, our models may be of particular interest to systematic reviewers and expert panels who want to quickly obtain relevant studies from among those not yet fully indexed in clinical literature databases. Databases such as PubMed may consider the deployment of such models in similar fashions as HIRU and Health Evidence or in conjunction with existing systems to improve the reliability of paper classifications.

Ultimately, organizations should carefully define the intended role of such models (ie, whether the models are used for initial classification for efficiency gains or deployed to inform human annotators to improve accuracy), as well as establish acceptable trade-offs between error rates, efficiency gains, and cost. Although patient privacy and safety are unlikely to be a concern, copyright issues may be a consideration for full-text use during model training and validation [90]. It is also crucial to train annotators in the effective interpretation of model outputs and responsible artificial intelligence (AI) use. Finally, our models may not be directly transferable to other databases and systematic review systems with different purposes. Nevertheless, insights from this work regarding model fine-tuning and selection are valuable for future development and benchmarking studies of similar scope.

### Comparison With Previous Work

To the best of our knowledge, no other experiment has used a similar corpus for multiclass classification. Therefore, direct comparisons of model performance were not feasible. We instead provide an overview of previous related studies.

Rabby and Berka [58] examined various shallow learning (SL) and deep learning (DL) methods to classify COVID-19 papers that had only one label in the LitCovid corpus and included studies in the prevention, treatment, diagnosis, or case report classes. Interestingly, the random forest classifier obtained the best performance, with 0.92 accuracy and macro *F*_1_-score using the term frequency–inverse document frequency (TF-IDF) as a feature. BERT, with an LR of 1 × 10^–5^, an epoch of 5, and a maximum token length of 256, achieved a lower performance of 0.87 accuracy and macro *F*_1_-score. We suspect that this could be due to insufficient training data, poor hyperparameter choices, or bias from the lack of a validation set for hyperparameter tuning.

Afzal et al [57] used SciBERT and the Unified Medical Language System (UMLS), with bidirectional long short‑term memory (BiLSTM) and active learning, to classify medical notes with the SOAP (Subjective, Objective, Assessment, Plan) structure. Specifically, SciBERT and the UMLS were used to process contextual and semantic information, respectively, before being combined and processed with a dense SoftMax layer. The system was named SOAP-BiomedBERT and achieved an accuracy of 0.98 and an *F*_1_-score of 0.99. Compared to SciBERT alone, SOAP-BiomedBERT represents a mild increase of ~0.01 in both accuracy and *F*_1_-scores.

Raja et al [91] used Bidirectional and Auto-Regressive Transformers (BART) to classify 1000 papers on retinal diseases into 4 classes and 18 labels. BART, as opposed to BERT, is a sequence-to-sequence model that incorporates both an encoder and a decoder. During the training process, inputs are corrupted by masking, deletion, and/or permutation, and the model learns to reconstruct the original text. The architecture makes it more effective at generative and transformation tasks compared to BERT. Classifying for study types (clinical, experimental, or automated), the model achieved an *F*_1_-score of 0.92 and an AUROC of 0.91. Although the performance has room for improvement, it was achieved through zero-shot learning without the need for an extensive annotated training set.

Joshi and Abdelfattah [92] explored the applicability of six SL multinomial classifiers to predict medical conditions from drug reviews. TF-IDF vectors were used as features, and a random search was used for hyperparameter tuning. The best-performing model, the linear support vector classifier, achieved an *F*_1_-score of 0.88. Naive Bayes, in contrast, performed the worst, with a score of 0.64, despite being the fastest model.

### Limitations and Future Directions

Several limitations must be noted. First, we were unable to use the full text of papers as inputs due to the intrinsic token limitations of BERT and ELECTRA, although the models performed well on titles and abstracts alone. Experiments using Longformer or other architectures that use the sliding window attention may be warranted. Generative large language models may be worth exploring as well, considering their task-agnostic nature and lower requirement for technical knowledge.

Second, cross-validation and nested cross-validation were not implemented due to computational and storage constraints. The number of papers included in this experiment should effectively reduce the risk of sampling bias. However, studies using a smaller dataset may wish to consider cross-validation during hyperparameter tuning to improve the reliability of the models.

Third, we only attempted CW adjustments to address class imbalance in this work, which did not improve the performance for the minority classes except in terms of recall. We did not explore other methods for comparison, such as other cost-sensitive learning methods; data augmentation, such as synonym replacement or undersampling; and hierarchical classification and ensemble schemes. This is primarily due to resource limitations, considering the large hyperparameter search space we examined. Although the models’ performance on minority classes remained strong, with AUROC≥99% and MCC≥0.80, we nevertheless believe that future studies using a more refined hyperparameter search informed by our results are warranted.

Lastly, all papers used for training were retrieved from clinically focused journals indexed in PubMed. Considering the performance degradation on Clinical Hedges, it is unknown how well the model would generalize to contemporary papers from other commonly used biomedical databases, such as Embase or Scopus, which have broader disciplinary scopes, or from databases with more specific focuses, such as Emcare and PsycINFO, which emphasize allied health, nursing, or behavioral sciences. Differences in subject focus, indexing practices, and paper composition may pose domain-specific challenges. Before external deployment, benchmarking model performance on these sources would be necessary. Applying the model to such databases would also likely require fine-tuning on representative samples from those sources, although this is hindered by the lack of publicly available datasets. Nevertheless, our methods could be readily replicated to achieve similar performance in those contexts.

As future work, the explainability of the models will be explored. One approach is to use integrated gradients or Shapley Additive Explanations (SHAP), which allow for feature attributions to be determined at the token level [93-95]. This means that the words (or tokens) that are most important to the model classification decision can be identified and shared with human assessors. Alignment of these words with human understanding of the task can improve the transparency and, thus, acceptance and adoption of AI models in supporting literature assessment.

### Conclusion

This study demonstrates the effectiveness of fine-tuning pretrained transformer models for multiclass classification of clinical literature, achieving high performance across most metrics and providing a practical solution for early classification of study types within a knowledge translation workflow. Among the 10 top-performing models, BioBERT variants exhibited superior calibration and consistent results, which could make them well suited for piloting in human-in-the-loop systems to support evidence synthesis workflows. Challenges, such as class imbalance and performance degradation on underrepresented classes (eg, evidence-based guidelines and nonexperimental studies), underscore the need for further exploration of hierarchical classification and strategies to handle rarer classes. Optimal configurations, including lower LRs, midrange BSs, and lower WDs, were critical to achieving robust performance, while CW adjustments proved less beneficial except for recall-focused metrics. These findings contribute to the growing body of research on automated text classification and hold promise for improving the efficiency of clinical literature curation and systematic reviews.

## Appendix

Multimedia Appendix 1 Evaluation metric, model training environment, best-performing models, performance of top models, aggregated model performance, confusion matrices ,and calibration plots.

Multimedia Appendix 2 Macro averages of evaluation metrics on the PLUS-validate set by model configuration parameters and performance of the top models on AUROC, *F*_1_-score, and MCC. AUROC: area under the receiver operating characteristic curve; MCC: Matthew’s correlation coefficient; PLUS: Premium Literature Service.

## Abbreviations

AI: artificial intelligence
AP: average precision
AUROC: area under the receiver operating characteristic curve
BART: Bidirectional and Auto-Regressive Transformers
BERT: Bidirectional Encoder Representations from Transformers
BioBERT: Biomedical Bidirectional Encoder Representations from Transformers (fine-tuned on biomedical text)
BioELECTRA: Biomedical Efficiently Learning an Encoder that Classifies Token Replacements Accurately
BioLinkBERT: Biomedical Document Link Bidirectional Encoder Representations from Transformers
BiomedBERT: Biomedical Bidirectional Encoder Representations from Transformers (trained entirely on biomedical text)
BS: batch size
CW: class weight
GPU: graphics processing unit
GRU: Gated recurrent unit
HIRU: Health Information Research Unit
LR: learning rate
MCC: Matthew’s correlation coefficient
MeSH: Medical Subject Headings
ML: machine learning
OvO: one versus one
OvR: one versus rest
PLUS: Premium Literature Service
SciBERT: Scientific Bidirectional Encoder Representations from Transformers
SL: shallow learning
SOAP: Subjective Objective Assessment Plan
TF-IDF: term frequency–inverse document frequency
UMLS: Unified Medical Language System
WD: weight decay
WR: warmup ratio

## Data Availability

All data and the source code are available upon reasonable request to the first or the corresponding author.
