# Supplementary material for: Fine-Tuning and Benchmarking Transformer Models for Multiclass Classification of Clinical Research Papers: Retrospective Modeling Study
Source: JMIR AI. 2026 Apr 29;5:e77311. doi: 10.2196/77311 (PMC13173073; doi:10.2196/77311)

**Supplementary Information**

[**Table S1.** Evaluation metric description 3](#__RefHeading___Toc193443474)

[**Table S2.** Model training environment 4](#__RefHeading___Toc193443475)

[**Table S3.** Configurations of the best performing models on the validation set 7](#__RefHeading___Toc193443476)

[**Table S4.** Performance of the top models on PLUS-validate 8](#__RefHeading___Toc193443477)

[**Table S5.** Performance of the top models on PLUS-test 12](#__RefHeading___Toc193443478)

[**Table S6.** Performance of the top models on Clinical Hedges 16](#__RefHeading___Toc193443479)

[**Figure S1.** Aggregated model performance on PLUS-validate by pretrained model 19](#__RefHeading___Toc193443480)

[**Figure S2.** Aggregated model performance on PLUS-validate by class weight adjustment 20](#__RefHeading___Toc193443481)

[**Figure S3.** Aggregated model performance on PLUS-validate by learning rate 21](#__RefHeading___Toc193443482)

[**Figure S4.** Aggregated model performance on PLUS-validate by batch size 22](#__RefHeading___Toc193443483)

[**Figure S5.** Aggregated model performance on PLUS-validate by warmup ratio 23](#__RefHeading___Toc193443484)

[**Figure S6.** Aggregated model performance on PLUS-validate by weight decay 24](#__RefHeading___Toc193443485)

[**Figure S7.** Confusion matrices for the best cross entropy loss model (BioBERT; CW: No; LR: 5E-5; BS: 256; WR: 0.10; WD: 0.015) 25](#__RefHeading___Toc193443486)

[**Figure S8.** Confusion matrices for the best Brier score model (BioBERT; CW: No; LR: 1E-5; BS: 64; WR: 0.20; WD: 0.015) 26](#__RefHeading___Toc193443487)

[**Figure S9.** Confusion matrices for the best average precision model (BiomedBERT; CW: No; LR: 1E-5; BS: 128; WR: 0.05; WD: 0.010) 27](#__RefHeading___Toc193443488)

[**Figure S10.** Confusion matrices for the best recall model (SciBERT-uncased; CW: Yes; LR: 3E-5; BS: 256; WR: 0.05; WD: 0.010) 28](#__RefHeading___Toc193443489)

[**Figure S11.** Confusion matrices for the best precision model (BioLinkBERT; CW: No; LR: 3E-5; BS: 16; WR: 0.10; WD: 0.010) 29](#__RefHeading___Toc193443490)

[**Figure S12.** Confusion matrices for the best accuracy model (BioBERT; CW: No; LR: 1E-5; BS: 256; WR: 0.05; WD: 0.015) 30](#__RefHeading___Toc193443491)

[**Figure S12.** Confusion matrices for the best F2 model (BiomedBERT; CW: Yes; LR: 3E-5; BS: 128; WR: 0.05; WD: 0.015) 31](#__RefHeading___Toc193443492)

[**Figure S13.** Calibration plots for the best cross entropy loss model (BioBERT; CW: No; LR: 5E-5; BS: 256; WR: 0.10; WD: 0.015) 32](#__RefHeading___Toc193443493)

[**Figure S14.** Calibration plots for the best Brier score model (BioBERT; CW: No; LR: 1E-5; BS: 64; WR: 0.20; WD: 0.015) 33](#__RefHeading___Toc193443494)

[**Figure S15.** Calibration plots for the best average precision model (BiomedBERT; CW: No; LR: 1E-5; BS: 128; WR: 0.05; WD: 0.010) 34](#__RefHeading___Toc193443495)

[**Figure S16.** Calibration plots for the best recall model (SciBERT-uncased; CW: Yes; LR: 3E-5; BS: 256; WR: 0.05; WD: 0.010) 35](#__RefHeading___Toc193443496)

[**Figure S17.** Calibration plots for the best precision model (BioLinkBERT; CW: No; LR: 3E-5; BS: 16; WR: 0.10; WD: 0.010) 36](#__RefHeading___Toc193443497)

[**Figure S18.** Calibration plots for the best accuracy model (BioBERT; CW: No; LR: 1E-5; BS: 256; WR: 0.05; WD: 0.015) 37](#__RefHeading___Toc193443498)

[**Figure S19.** Calibration plots for the best F2 model (BiomedBERT; CW: Yes; LR: 3E-5; BS: 128; WR: 0.05; WD: 0.015) 38](#__RefHeading___Toc193443499)

# **Table S1.** Evaluation metric description

| **Metric** | **Definition** | **Formula** | **Range** |
| --- | --- | --- | --- |
| Cross entropy loss/log loss | Mean log difference between predicted probability and true probability. | -- | 0 to infinity; lower is better |
| Brier score | Mean squared difference between predicted probability and true probability. | -- | 0 to 1; lower is better |
| AUROC | The area under the receiver operating characteristic curve; the ability to maintain specificity as sensitivity increases. | -- | 0 to 1; higher is better |
| AP | Weighted mean of precisions achieved at each threshold where recall changes; the ability to maintain precision as recall increases. | -- | 0 to 1; higher is better |
| Recall | The proportion of correctly classified positive instances. | 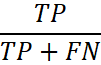 | 0 to 1; higher is better |
| Accuracy | The proportion of correct classifications. | 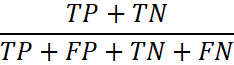 | 0 to 1; higher is better |
| Precision | The proportion of correct positive classifications. | 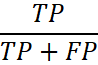 | 0 to 1; higher is better |
| F1 | The harmonic mean of precision and recall. | 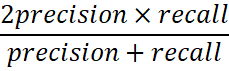 | 0 to 1; higher is better |
| F2 | The harmonic mean of precision and recall with an emphasis on recall. | 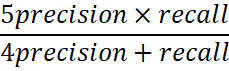 | 0 to 1; higher is better |
| MCC | The quality of the classifications with a given probability threshold in considerations of sensitivity, specificity, precision, and NPV. | 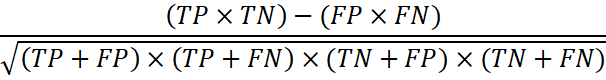 | -1 to 1; higher is better |

**AP** Average precision; **AUROC** Area under the receiver operating characteristic curve; Average precision; **FN** False negative; **FP** False positive; **MCC** Matthews correlation coefficient; **ROC** Receiver operating characteristic; **TN** True negative; **TP** True positive.

# **Table S2.** Model training environment

| **Library** | **Version** |
| --- | --- |
| accelerate | 0.27.2+computecanada |
| aiohttp | 3.9.1+computecanada |
| aiosignal | 1.3.1+computecanada |
| anyio | 3.7.1+computecanada |
| appdirs | 1.4.4+computecanada |
| arff | 0.9+computecanada |
| argon2_cffi | 23.1.0+computecanada |
| argon2_cffi_bindings | 21.2.0+computecanada |
| asttokens | 2.2.1+computecanada |
| async_generator | 1.10+computecanada |
| attrs | 23.1.0+computecanada |
| backcall | 0.2.0+computecanada |
| backports-abc | 0.5+computecanada |
| backports.shutil_get_terminal_size | 1.0.0+computecanada |
| bcrypt | 4.0.1+computecanada |
| beautifulsoup4 | 4.12.2+computecanada |
| bitarray | 2.8.1+computecanada |
| bitstring | 4.1.1+computecanada |
| bleach | 6.0.0+computecanada |
| certifi | 2023.7.22+computecanada |
| cffi | 1.15.1+computecanada |
| chardet | 5.2.0+computecanada |
| charset_normalizer | 3.2.0+computecanada |
| click | 8.1.7+computecanada |
| comm | 0.1.4+computecanada |
| contourpy | 1.1.0+computecanada |
| cryptography | 39.0.1+computecanada |
| cycler | 0.11.0+computecanada |
| Cython | 0.29.36+computecanada |
| datasets | 2.18.0+computecanada |
| deap | 1.4.1+computecanada |
| debugpy | 1.6.7.post1+computecanada |
| decorator | 5.1.1+computecanada |
| defusedxml | 0.7.1+computecanada |
| dill | 0.3.8+computecanada |
| dnspython | 2.4.2+computecanada |
| docker-pycreds | 0.4.0+computecanada |
| ecdsa | 0.18.0+computecanada |
| entrypoints | 0.4+computecanada |
| evaluate | 0.4.2+computecanada |
| executing | 1.2.0+computecanada |
| fastjsonschema | 2.18.0+computecanada |
| filelock | 3.13.1+computecanada |
| fonttools | 4.42.1+computecanada |
| frozenlist | 1.4.1+computecanada |
| fsspec | 2024.2.0+computecanada |
| funcsigs | 1.0.2+computecanada |
| gitdb | 4.0.11+computecanada |
| GitPython | 3.1.40+computecanada |
| huggingface_hub | 0.21.4+computecanada |
| idna | 3.4+computecanada |
| importlib_metadata | 6.8.0+computecanada |
| importlib_resources | 6.0.1+computecanada |
| ipykernel | 6.25.1+computecanada |
| ipython | 8.15.0+computecanada |
| ipython_genutils | 0.2.0+computecanada |
| jedi | 0.19.0+computecanada |
| Jinja2 | 3.1.2+computecanada |
| joblib | 1.3.2+computecanada |
| jsonschema | 4.19.0+computecanada |
| jsonschema_specifications | 2023.7.1+computecanada |
| jupyter_client | 8.3.1+computecanada |
| jupyter_core | 5.3.1+computecanada |
| kiwisolver | 1.4.5+computecanada |
| lockfile | 0.12.2+computecanada |
| MarkupSafe | 2.1.3+computecanada |
| matplotlib | 3.7.2+computecanada |
| matplotlib_inline | 0.1.6+computecanada |
| mistune | 3.0.1+computecanada |
| mock | 5.1.0+computecanada |
| mpmath | 1.3.0+computecanada |
| multidict | 6.0.5+computecanada |
| multiprocess | 0.70.16+computecanada |
| nest_asyncio | 1.5.7+computecanada |
| netaddr | 0.8.0+computecanada |
| netifaces | 0.11.0+computecanada |
| networkx | 3.2.1+computecanada |
| nose | 1.3.7+computecanada |
| numpy | 1.25.2+computecanada |
| packaging | 23.1+computecanada |
| pandas | 2.1.0+computecanada |
| pandocfilters | 1.5.0+computecanada |
| paramiko | 3.3.1+computecanada |
| parso | 0.8.3+computecanada |
| path | 16.7.1+computecanada |
| path.py | 12.5.0+computecanada |
| pathlib2 | 2.3.7.post1+computecanada |
| paycheck | 1.0.2+computecanada |
| pbr | 5.11.1+computecanada |
| pexpect | 4.8.0+computecanada |
| pickleshare | 0.7.5+computecanada |
| Pillow | 10.0.0+computecanada |
| pkgutil_resolve_name | 1.3.10+computecanada |
| platformdirs | 3.9.1+computecanada |
| prometheus_client | 0.17.1+computecanada |
| prompt_toolkit | 3.0.39+computecanada |
| protobuf | 4.25.2+computecanada |
| psutil | 5.9.5+computecanada |
| ptyprocess | 0.7.0+computecanada |
| pure_eval | 0.2.2+computecanada |
| pyarrow | 15.0.1 |
| pyarrow_hotfix | 0.6+computecanada |
| pycparser | 2.21+computecanada |
| Pygments | 2.16.1+computecanada |
| PyNaCl | 1.5.0+computecanada |
| pyparsing | 3.0.9+computecanada |
| pyrsistent | 0.19.3+computecanada |
| python-dateutil | 2.8.2+computecanada |
| python_json_logger | 2.0.7+computecanada |
| pytz | 2023.3+computecanada |
| PyYAML | 6.0.1+computecanada |
| pyzmq | 25.1.1+computecanada |
| referencing | 0.30.2+computecanada |
| regex | 2023.8.8+computecanada |
| requests | 2.31.0+computecanada |
| responses | 0.18.0+computecanada |
| rfc3339_validator | 0.1.4+computecanada |
| rfc3986_validator | 0.1.1+computecanada |
| rpds_py | 0.10.0+computecanada |
| safetensors | 0.4.1+computecanada |
| scikit_learn | 1.3.1+computecanada |
| scipy | 1.11.2+computecanada |
| Send2Trash | 1.8.2+computecanada |
| sentry_sdk | 1.38.0+computecanada |
| setproctitle | 1.3.2+computecanada |
| shap | 0.43.0+computecanada |
| simplegeneric | 0.8.1+computecanada |
| singledispatch | 4.1.0+computecanada |
| six | 1.16.0+computecanada |
| sklearn | 0.0+computecanada |
| smmap | 5.0.1+computecanada |
| sniffio | 1.3.0+computecanada |
| soupsieve | 2.4.1+computecanada |
| stack_data | 0.6.2+computecanada |
| sympy | 1.12+computecanada |
| terminado | 0.17.1+computecanada |
| testpath | 0.6.0+computecanada |
| threadpoolctl | 3.3.0+computecanada |
| tinycss2 | 1.2.1+computecanada |
| tokenizers | 0.15.0+computecanada |
| torch | 2.2.1+computecanada |
| tornado | 6.3.3+computecanada |
| tqdm | 4.66.2+computecanada |
| traitlets | 5.9.0+computecanada |
| transformers | 4.38.1+computecanada |
| typing_extensions | 4.10.0+computecanada |
| tzdata | 2023.3+computecanada |
| urllib3 | 2.0.4+computecanada |
| wandb | 0.16.0+computecanada |
| wcwidth | 0.2.6+computecanada |
| webencodings | 0.5.1+computecanada |
| websocket_client | 1.6.2+computecanada |
| xxhash | 3.2.0+computecanada |
| yarl | 1.9.3+computecanada |
| zipp | 3.16.2+computecanada |

# **Table S3.** Configurations of the best performing models on the validation set

| **Best Metric** | **Model** | **Class Weights** | **Learning Rate** | **Batch Size** | **Warmup Ratio** | **Weight Decay** |
| --- | --- | --- | --- | --- | --- | --- |
| Cross Entropy Loss | BioBERT | No | 5E-5 | 256 | 0.10 | 0.015 |
| Brier Score | BioBERT | No | 1E-5 | 64 | 0.20 | 0.015 |
| AUROC | BiomedBERT | No | 3E-5 | 256 | 0.20 | 0.005 |
| AUPRC | BiomedBERT | No | 1E-5 | 128 | 0.05 | 0.010 |
| Recall | SciBERT-uncased | Yes | 3E-5 | 256 | 0.05 | 0.010 |
| Precision | BioLinkBERT | No | 3E-5 | 16 | 0.10 | 0.010 |
| Accuracy | BioBERT | No | 1E-5 | 256 | 0.05 | 0.015 |
| F1 | BioBERT | No | 5E-5 | 64 | 0.10 | 0.015 |
| F2 | BiomedBERT | Yes | 3E-5 | 128 | 0.05 | 0.015 |
| MCC | BiomedBERT | No | 1E-5 | 16 | 0.20 | 0.005 |

# **Table S4.** Performance of the top models on PLUS-validate

| **Model (Best metric; CW, LR, BS, WR, WD)** | **Class** | **Cross-entropy Loss** | **Brier Score** | **AUROC** | **AUPRC** | **Recall** | **Precision** | **Accuracy** | **F1 Score** | **F2 Score** | **MCC** |
| --- | --- | --- | --- | --- | --- | --- | --- | --- | --- | --- | --- |
| BioBERT (Cross entropy loss; No, 5E-5, 256, 0.10, 0.015) | Original study | 0.048 (0.043, 0.053) | 0.012 (0.011, 0.013) | 0.998 (0.997, 0.998) | 0.999 (0.998, 0.999) | 0.985 (0.982, 0.987) | 0.992 (0.990, 0.994) | 0.985 (0.983, 0.987) | 0.988 (0.987, 0.990) | 0.986 (0.984, 0.988) | 0.967 (0.962, 0.971) |
|  | Review | 0.052 (0.047, 0.057) | 0.015 (0.013, 0.016) | 0.997 (0.997, 0.998) | 0.992 (0.991, 0.994) | 0.966 (0.961, 0.972) | 0.952 (0.946, 0.959) | 0.980 (0.978, 0.982) | 0.959 (0.955, 0.963) | 0.963 (0.958, 0.968) | 0.946 (0.941, 0.952) |
|  | Evidence-based guideline | 0.017 (0.015, 0.020) | 0.005 (0.004, 0.006) | 0.996 (0.994, 0.998) | 0.903 (0.875, 0.929) | 0.870 (0.831, 0.907) | 0.807 (0.767, 0.853) | 0.994 (0.993, 0.995) | 0.837 (0.805, 0.870) | 0.857 (0.824, 0.888) | 0.835 (0.802, 0.868) |
|  | Non-experimental | 0.058 (0.054, 0.063) | 0.017 (0.015, 0.018) | 0.992 (0.990, 0.994) | 0.940 (0.930, 0.949) | 0.870 (0.853, 0.886) | 0.871 (0.853, 0.889) | 0.978 (0.975, 0.980) | 0.870 (0.856, 0.883) | 0.870 (0.855, 0.884) | 0.858 (0.843, 0.871) |
|  | Macro average | 0.044 (0.041, 0.047) | 0.012 (0.011, 0.013) | 0.996 (0.995, 0.997) | 0.959 (0.951, 0.966) | 0.923 (0.912, 0.933) | 0.906 (0.894, 0.918) | 0.984 (0.983, 0.985) | 0.914 (0.904, 0.923) | 0.919 (0.909, 0.928) | 0.901 (0.891, 0.911) |
| BioBERT (Brier score; No, 1E-5, 64, 0.20, 0.015) | Original study | 0.050 (0.044, 0.056) | 0.012 (0.010, 0.013) | 0.997 (0.997, 0.998) | 0.999 (0.998, 0.999) | 0.986 (0.984, 0.989) | 0.991 (0.990, 0.993) | 0.985 (0.984, 0.987) | 0.989 (0.987, 0.990) | 0.987 (0.985, 0.989) | 0.968 (0.964, 0.972) |
|  | Review | 0.053 (0.047, 0.058) | 0.014 (0.013, 0.016) | 0.997 (0.997, 0.998) | 0.992 (0.991, 0.994) | 0.961 (0.955, 0.967) | 0.960 (0.954, 0.966) | 0.981 (0.979, 0.983) | 0.960 (0.956, 0.965) | 0.961 (0.956, 0.966) | 0.948 (0.942, 0.954) |
|  | Evidence-based guideline | 0.017 (0.014, 0.020) | 0.005 (0.004, 0.005) | 0.995 (0.992, 0.998) | 0.898 (0.867, 0.926) | 0.833 (0.792, 0.874) | 0.827 (0.784, 0.874) | 0.994 (0.993, 0.995) | 0.830 (0.799, 0.863) | 0.832 (0.798, 0.867) | 0.827 (0.796, 0.861) |
|  | Non-experimental | 0.060 (0.054, 0.066) | 0.017 (0.015, 0.018) | 0.991 (0.988, 0.993) | 0.936 (0.924, 0.947) | 0.889 (0.873, 0.905) | 0.857 (0.840, 0.877) | 0.978 (0.975, 0.980) | 0.873 (0.861, 0.887) | 0.883 (0.869, 0.897) | 0.861 (0.847, 0.876) |
|  | Macro average | 0.045 (0.041, 0.049) | 0.012 (0.011, 0.013) | 0.995 (0.994, 0.996) | 0.956 (0.948, 0.964) | 0.917 (0.907, 0.928) | 0.909 (0.898, 0.922) | 0.984 (0.983, 0.986) | 0.913 (0.904, 0.923) | 0.916 (0.906, 0.926) | 0.901 (0.892, 0.912) |
| BiomedBERT (AUROC; No, 3E-5, 256, 0.20, 0.005) | Original study | 0.049 (0.043, 0.055) | 0.012 (0.011, 0.014) | 0.998 (0.997, 0.998) | 0.999 (0.998, 0.999) | 0.988 (0.985, 0.990) | 0.989 (0.987, 0.991) | 0.985 (0.983, 0.987) | 0.988 (0.987, 0.990) | 0.988 (0.986, 0.990) | 0.966 (0.962, 0.971) |
|  | Review | 0.053 (0.048, 0.058) | 0.015 (0.013, 0.016) | 0.997 (0.997, 0.998) | 0.993 (0.991, 0.994) | 0.962 (0.955, 0.968) | 0.957 (0.951, 0.964) | 0.980 (0.978, 0.983) | 0.959 (0.955, 0.964) | 0.961 (0.956, 0.966) | 0.947 (0.940, 0.953) |
|  | Evidence-based guideline | 0.016 (0.013, 0.019) | 0.004 (0.004, 0.005) | 0.997 (0.996, 0.998) | 0.907 (0.876, 0.934) | 0.843 (0.801, 0.881) | 0.832 (0.788, 0.874) | 0.994 (0.993, 0.995) | 0.837 (0.804, 0.868) | 0.841 (0.804, 0.873) | 0.834 (0.800, 0.866) |
|  | Non-experimental | 0.059 (0.054, 0.065) | 0.017 (0.015, 0.019) | 0.992 (0.990, 0.994) | 0.941 (0.932, 0.950) | 0.867 (0.850, 0.885) | 0.868 (0.850, 0.885) | 0.977 (0.975, 0.979) | 0.867 (0.854, 0.880) | 0.867 (0.852, 0.882) | 0.855 (0.840, 0.869) |
|  | Macro average | 0.044 (0.041, 0.048) | 0.012 (0.011, 0.013) | 0.996 (0.995, 0.997) | 0.960 (0.951, 0.967) | 0.915 (0.903, 0.926) | 0.912 (0.899, 0.923) | 0.984 (0.983, 0.985) | 0.913 (0.903, 0.923) | 0.914 (0.903, 0.924) | 0.901 (0.890, 0.911) |
| BiomedBERT (AUPRC; No, 1E-5, 128, 0.05, 0.010) | Original study | 0.051 (0.044, 0.057) | 0.012 (0.011, 0.014) | 0.997 (0.997, 0.998) | 0.998 (0.997, 0.999) | 0.988 (0.986, 0.990) | 0.988 (0.986, 0.990) | 0.984 (0.982, 0.986) | 0.988 (0.986, 0.989) | 0.988 (0.986, 0.990) | 0.965 (0.960, 0.969) |
|  | Review | 0.054 (0.049, 0.060) | 0.015 (0.014, 0.017) | 0.997 (0.997, 0.998) | 0.993 (0.991, 0.994) | 0.966 (0.960, 0.972) | 0.949 (0.942, 0.955) | 0.979 (0.977, 0.982) | 0.957 (0.953, 0.962) | 0.963 (0.958, 0.967) | 0.944 (0.938, 0.950) |
|  | Evidence-based guideline | 0.017 (0.014, 0.020) | 0.005 (0.004, 0.005) | 0.997 (0.996, 0.998) | 0.910 (0.883, 0.935) | 0.833 (0.792, 0.878) | 0.824 (0.776, 0.864) | 0.994 (0.993, 0.995) | 0.829 (0.794, 0.861) | 0.831 (0.796, 0.868) | 0.825 (0.791, 0.858) |
|  | Non-experimental | 0.061 (0.056, 0.067) | 0.017 (0.016, 0.019) | 0.992 (0.990, 0.994) | 0.940 (0.930, 0.950) | 0.837 (0.817, 0.857) | 0.885 (0.868, 0.902) | 0.976 (0.974, 0.979) | 0.860 (0.846, 0.874) | 0.846 (0.829, 0.863) | 0.848 (0.832, 0.863) |
|  | Macro average | 0.046 (0.042, 0.049) | 0.012 (0.011, 0.013) | 0.996 (0.995, 0.997) | 0.960 (0.953, 0.967) | 0.906 (0.894, 0.918) | 0.911 (0.899, 0.923) | 0.983 (0.982, 0.985) | 0.908 (0.898, 0.918) | 0.907 (0.896, 0.918) | 0.895 (0.884, 0.906) |
| SciBERT-uncased (Recall; Yes, 3E-5, 256, 0.05, 0.010) | Original study | 0.070 (0.063, 0.078) | 0.016 (0.015, 0.018) | 0.997 (0.997, 0.998) | 0.998 (0.998, 0.999) | 0.975 (0.973, 0.978) | 0.996 (0.995, 0.997) | 0.981 (0.979, 0.983) | 0.985 (0.984, 0.987) | 0.979 (0.977, 0.982) | 0.959 (0.955, 0.964) |
|  | Review | 0.065 (0.059, 0.071) | 0.017 (0.016, 0.019) | 0.997 (0.996, 0.997) | 0.990 (0.988, 0.992) | 0.942 (0.934, 0.948) | 0.967 (0.961, 0.972) | 0.978 (0.976, 0.980) | 0.954 (0.949, 0.958) | 0.946 (0.940, 0.952) | 0.940 (0.933, 0.945) |
|  | Evidence-based guideline | 0.028 (0.024, 0.033) | 0.008 (0.007, 0.009) | 0.996 (0.994, 0.998) | 0.899 (0.871, 0.926) | 0.939 (0.909, 0.962) | 0.655 (0.610, 0.701) | 0.990 (0.989, 0.992) | 0.771 (0.737, 0.805) | 0.864 (0.838, 0.889) | 0.779 (0.749, 0.811) |
|  | Non-experimental | 0.082 (0.076, 0.088) | 0.022 (0.021, 0.024) | 0.988 (0.986, 0.990) | 0.919 (0.907, 0.929) | 0.908 (0.894, 0.922) | 0.799 (0.779, 0.818) | 0.972 (0.970, 0.975) | 0.850 (0.836, 0.863) | 0.883 (0.871, 0.896) | 0.836 (0.822, 0.851) |
|  | Macro average | 0.061 (0.057, 0.066) | 0.016 (0.015, 0.017) | 0.995 (0.994, 0.995) | 0.952 (0.944, 0.959) | 0.941 (0.933, 0.948) | 0.854 (0.842, 0.866) | 0.980 (0.979, 0.982) | 0.890 (0.880, 0.901) | 0.918 (0.910, 0.926) | 0.879 (0.868, 0.889) |
| BioLinkBERT (Precision; No, 3E-5, 16, 0.10, 0.010) | Original study | 0.061 (0.053, 0.069) | 0.014 (0.012, 0.015) | 0.996 (0.996, 0.997) | 0.998 (0.998, 0.999) | 0.987 (0.985, 0.989) | 0.989 (0.987, 0.991) | 0.985 (0.983, 0.987) | 0.988 (0.987, 0.990) | 0.988 (0.986, 0.989) | 0.966 (0.962, 0.970) |
|  | Review | 0.061 (0.055, 0.068) | 0.017 (0.015, 0.018) | 0.997 (0.997, 0.998) | 0.992 (0.990, 0.993) | 0.967 (0.961, 0.972) | 0.946 (0.939, 0.953) | 0.979 (0.977, 0.981) | 0.956 (0.952, 0.961) | 0.963 (0.958, 0.967) | 0.942 (0.937, 0.948) |
|  | Evidence-based guideline | 0.024 (0.019, 0.029) | 0.005 (0.004, 0.006) | 0.994 (0.991, 0.997) | 0.890 (0.855, 0.920) | 0.805 (0.762, 0.848) | 0.871 (0.829, 0.911) | 0.994 (0.993, 0.995) | 0.837 (0.804, 0.868) | 0.818 (0.778, 0.854) | 0.835 (0.802, 0.866) |
|  | Non-experimental | 0.077 (0.069, 0.085) | 0.019 (0.017, 0.021) | 0.990 (0.988, 0.992) | 0.927 (0.914, 0.940) | 0.853 (0.834, 0.870) | 0.880 (0.863, 0.898) | 0.977 (0.975, 0.979) | 0.866 (0.853, 0.880) | 0.858 (0.842, 0.873) | 0.854 (0.839, 0.868) |
|  | Macro average | 0.056 (0.051, 0.061) | 0.014 (0.013, 0.015) | 0.995 (0.993, 0.996) | 0.952 (0.942, 0.960) | 0.903 (0.891, 0.915) | 0.922 (0.910, 0.933) | 0.984 (0.982, 0.985) | 0.912 (0.902, 0.921) | 0.907 (0.895, 0.917) | 0.899 (0.889, 0.910) |
| BioBERT (Accuracy; No, 1E-5, 256, 0.05, 0.015) | Original study | 0.049 (0.043, 0.056) | 0.012 (0.010, 0.013) | 0.997 (0.997, 0.998) | 0.998 (0.998, 0.999) | 0.986 (0.984, 0.988) | 0.991 (0.990, 0.993) | 0.985 (0.984, 0.987) | 0.989 (0.987, 0.990) | 0.987 (0.985, 0.989) | 0.968 (0.964, 0.972) |
|  | Review | 0.052 (0.046, 0.057) | 0.014 (0.013, 0.016) | 0.998 (0.997, 0.998) | 0.993 (0.991, 0.994) | 0.964 (0.957, 0.969) | 0.959 (0.953, 0.966) | 0.981 (0.979, 0.984) | 0.961 (0.957, 0.966) | 0.963 (0.958, 0.967) | 0.949 (0.943, 0.955) |
|  | Evidence-based guideline | 0.017 (0.014, 0.020) | 0.005 (0.004, 0.005) | 0.996 (0.992, 0.998) | 0.896 (0.865, 0.925) | 0.829 (0.783, 0.871) | 0.818 (0.774, 0.861) | 0.994 (0.992, 0.995) | 0.824 (0.789, 0.856) | 0.827 (0.787, 0.863) | 0.820 (0.786, 0.853) |
|  | Non-experimental | 0.060 (0.054, 0.066) | 0.017 (0.015, 0.018) | 0.991 (0.988, 0.993) | 0.936 (0.924, 0.946) | 0.886 (0.872, 0.903) | 0.865 (0.846, 0.882) | 0.978 (0.976, 0.980) | 0.875 (0.862, 0.888) | 0.882 (0.868, 0.896) | 0.863 (0.849, 0.877) |
|  | Macro average | 0.045 (0.041, 0.048) | 0.012 (0.011, 0.013) | 0.995 (0.994, 0.996) | 0.956 (0.947, 0.964) | 0.916 (0.905, 0.928) | 0.908 (0.897, 0.919) | 0.985 (0.983, 0.986) | 0.912 (0.903, 0.922) | 0.915 (0.904, 0.925) | 0.900 (0.890, 0.910) |
| BioBERT (F1; No, 5E-5, 64, 0.10, 0.015) | Original study | 0.051 (0.045, 0.058) | 0.012 (0.011, 0.014) | 0.997 (0.997, 0.998) | 0.998 (0.997, 0.999) | 0.986 (0.984, 0.988) | 0.991 (0.989, 0.993) | 0.985 (0.983, 0.987) | 0.988 (0.987, 0.990) | 0.987 (0.985, 0.989) | 0.967 (0.963, 0.971) |
|  | Review | 0.058 (0.052, 0.064) | 0.015 (0.014, 0.017) | 0.997 (0.997, 0.998) | 0.992 (0.991, 0.994) | 0.959 (0.952, 0.965) | 0.961 (0.955, 0.967) | 0.981 (0.979, 0.983) | 0.960 (0.956, 0.964) | 0.959 (0.954, 0.964) | 0.947 (0.942, 0.953) |
|  | Evidence-based guideline | 0.019 (0.015, 0.023) | 0.005 (0.004, 0.006) | 0.996 (0.993, 0.997) | 0.889 (0.853, 0.919) | 0.843 (0.799, 0.884) | 0.829 (0.787, 0.869) | 0.994 (0.993, 0.995) | 0.836 (0.801, 0.867) | 0.840 (0.801, 0.875) | 0.833 (0.798, 0.864) |
|  | Non-experimental | 0.062 (0.056, 0.068) | 0.017 (0.016, 0.019) | 0.991 (0.989, 0.993) | 0.934 (0.923, 0.944) | 0.892 (0.874, 0.908) | 0.854 (0.835, 0.872) | 0.977 (0.975, 0.980) | 0.873 (0.860, 0.885) | 0.884 (0.869, 0.897) | 0.861 (0.847, 0.873) |
|  | Macro average | 0.048 (0.044, 0.051) | 0.012 (0.011, 0.013) | 0.995 (0.994, 0.996) | 0.953 (0.944, 0.962) | 0.920 (0.908, 0.931) | 0.909 (0.897, 0.920) | 0.984 (0.983, 0.986) | 0.914 (0.904, 0.924) | 0.918 (0.907, 0.927) | 0.902 (0.892, 0.912) |
| BiomedBERT (F2; Yes, 3E-5, 128, 0.05, 0.015) | Original study | 0.059 (0.052, 0.066) | 0.014 (0.012, 0.015) | 0.997 (0.997, 0.998) | 0.999 (0.998, 0.999) | 0.982 (0.980, 0.985) | 0.993 (0.992, 0.995) | 0.984 (0.982, 0.986) | 0.988 (0.986, 0.989) | 0.985 (0.982, 0.987) | 0.965 (0.961, 0.970) |
|  | Review | 0.057 (0.051, 0.063) | 0.015 (0.014, 0.017) | 0.997 (0.996, 0.998) | 0.990 (0.987, 0.993) | 0.950 (0.944, 0.957) | 0.966 (0.961, 0.972) | 0.980 (0.978, 0.982) | 0.958 (0.953, 0.963) | 0.954 (0.948, 0.959) | 0.945 (0.939, 0.951) |
|  | Evidence-based guideline | 0.025 (0.021, 0.030) | 0.006 (0.005, 0.007) | 0.995 (0.991, 0.998) | 0.887 (0.853, 0.918) | 0.901 (0.865, 0.932) | 0.744 (0.699, 0.791) | 0.993 (0.991, 0.994) | 0.815 (0.783, 0.847) | 0.864 (0.835, 0.892) | 0.815 (0.782, 0.847) |
|  | Non-experimental | 0.068 (0.062, 0.075) | 0.019 (0.017, 0.020) | 0.991 (0.989, 0.993) | 0.930 (0.919, 0.940) | 0.908 (0.894, 0.921) | 0.836 (0.818, 0.853) | 0.977 (0.974, 0.979) | 0.870 (0.857, 0.882) | 0.892 (0.880, 0.905) | 0.858 (0.844, 0.871) |
|  | Macro average | 0.052 (0.048, 0.056) | 0.013 (0.012, 0.014) | 0.995 (0.994, 0.996) | 0.952 (0.942, 0.960) | 0.935 (0.926, 0.944) | 0.885 (0.873, 0.898) | 0.983 (0.982, 0.985) | 0.908 (0.898, 0.917) | 0.924 (0.915, 0.932) | 0.896 (0.886, 0.906) |
| BiomedBERT (MCC; No, 1E-5, 16, 0.20, 0.005) | Original study | 0.063 (0.055, 0.071) | 0.013 (0.012, 0.015) | 0.997 (0.997, 0.998) | 0.998 (0.998, 0.999) | 0.986 (0.984, 0.988) | 0.991 (0.990, 0.993) | 0.985 (0.983, 0.987) | 0.989 (0.987, 0.990) | 0.987 (0.985, 0.989) | 0.967 (0.963, 0.972) |
|  | Review | 0.064 (0.057, 0.071) | 0.016 (0.014, 0.017) | 0.997 (0.996, 0.998) | 0.992 (0.990, 0.993) | 0.964 (0.958, 0.970) | 0.958 (0.951, 0.964) | 0.981 (0.979, 0.983) | 0.961 (0.956, 0.965) | 0.963 (0.958, 0.968) | 0.949 (0.943, 0.954) |
|  | Evidence-based guideline | 0.022 (0.018, 0.027) | 0.005 (0.004, 0.006) | 0.994 (0.991, 0.997) | 0.880 (0.839, 0.911) | 0.816 (0.772, 0.858) | 0.860 (0.820, 0.900) | 0.994 (0.993, 0.995) | 0.837 (0.805, 0.866) | 0.824 (0.786, 0.859) | 0.835 (0.802, 0.864) |
|  | Non-experimental | 0.072 (0.064, 0.079) | 0.019 (0.017, 0.021) | 0.991 (0.989, 0.993) | 0.934 (0.923, 0.943) | 0.883 (0.867, 0.900) | 0.857 (0.839, 0.875) | 0.977 (0.975, 0.979) | 0.870 (0.856, 0.883) | 0.878 (0.863, 0.892) | 0.857 (0.843, 0.872) |
|  | Macro average | 0.055 (0.050, 0.060) | 0.013 (0.012, 0.014) | 0.995 (0.994, 0.996) | 0.951 (0.940, 0.960) | 0.912 (0.901, 0.924) | 0.916 (0.905, 0.928) | 0.984 (0.983, 0.986) | 0.914 (0.904, 0.923) | 0.913 (0.903, 0.923) | 0.902 (0.892, 0.912) |

**AP** Average precision; **AUROC** Area under the receiver operating characteristic curve; **BS** Batch size; **CW** Class weights; **LR** Learning rate; **MCC** Matthews correlation coefficient; **WD** Weight decay; **WR** Warmup ratio.

**Note:** All values are presented as score (95% CI).

# **Table S5.** Performance of the top models on PLUS-test

| **Model (Best metric; CW, LR, BS, WR, WD)** | **Class** | **Cross-entropy Loss** | **Brier Score** | **AUROC** | **AUPRC** | **Recall** | **Precision** | **Accuracy** | **F1 Score** | **F2 Score** | **MCC** |
| --- | --- | --- | --- | --- | --- | --- | --- | --- | --- | --- | --- |
| BioBERT (Cross entropy loss; No, 5E-5, 256, 0.10, 0.015) | Original study | 0.052 (0.047, 0.058) | 0.013 (0.012, 0.014) | 0.997 (0.996, 0.998) | 0.998 (0.997, 0.999) | 0.985 (0.983, 0.988) | 0.990 (0.988, 0.992) | 0.984 (0.982, 0.986) | 0.988 (0.986, 0.989) | 0.986 (0.984, 0.988) | 0.965 (0.961, 0.969) |
|  | Review | 0.054 (0.048, 0.059) | 0.014 (0.013, 0.016) | 0.997 (0.996, 0.998) | 0.992 (0.990, 0.993) | 0.963 (0.958, 0.969) | 0.961 (0.955, 0.967) | 0.981 (0.979, 0.984) | 0.962 (0.958, 0.966) | 0.963 (0.958, 0.968) | 0.950 (0.944, 0.956) |
|  | Evidence-based guideline | 0.020 (0.017, 0.023) | 0.006 (0.005, 0.007) | 0.996 (0.994, 0.997) | 0.881 (0.849, 0.910) | 0.827 (0.788, 0.867) | 0.804 (0.759, 0.846) | 0.993 (0.991, 0.994) | 0.816 (0.781, 0.846) | 0.822 (0.786, 0.856) | 0.812 (0.777, 0.843) |
|  | Non-experimental | 0.060 (0.055, 0.066) | 0.017 (0.015, 0.018) | 0.991 (0.988, 0.993) | 0.940 (0.931, 0.948) | 0.886 (0.869, 0.902) | 0.869 (0.853, 0.886) | 0.978 (0.976, 0.980) | 0.877 (0.865, 0.889) | 0.882 (0.868, 0.896) | 0.866 (0.852, 0.878) |
|  | Macro average | 0.047 (0.043, 0.050) | 0.012 (0.012, 0.013) | 0.995 (0.994, 0.996) | 0.953 (0.944, 0.961) | 0.915 (0.904, 0.926) | 0.906 (0.894, 0.918) | 0.984 (0.983, 0.985) | 0.911 (0.900, 0.920) | 0.913 (0.902, 0.923) | 0.898 (0.887, 0.908) |
| BioBERT (Brier score; No, 1E-5, 64, 0.20, 0.015) | Original study | 0.056 (0.049, 0.064) | 0.013 (0.012, 0.015) | 0.997 (0.996, 0.997) | 0.998 (0.997, 0.999) | 0.985 (0.983, 0.988) | 0.990 (0.988, 0.992) | 0.984 (0.982, 0.986) | 0.987 (0.986, 0.989) | 0.986 (0.984, 0.988) | 0.965 (0.960, 0.969) |
|  | Review | 0.056 (0.050, 0.063) | 0.014 (0.013, 0.016) | 0.996 (0.995, 0.997) | 0.990 (0.987, 0.992) | 0.959 (0.953, 0.965) | 0.965 (0.960, 0.971) | 0.982 (0.979, 0.984) | 0.962 (0.958, 0.966) | 0.960 (0.955, 0.965) | 0.950 (0.945, 0.955) |
|  | Evidence-based guideline | 0.020 (0.017, 0.023) | 0.005 (0.004, 0.006) | 0.994 (0.991, 0.997) | 0.885 (0.856, 0.911) | 0.796 (0.754, 0.837) | 0.841 (0.801, 0.881) | 0.993 (0.992, 0.994) | 0.817 (0.787, 0.847) | 0.804 (0.768, 0.839) | 0.814 (0.783, 0.845) |
|  | Non-experimental | 0.061 (0.055, 0.066) | 0.017 (0.015, 0.018) | 0.990 (0.988, 0.993) | 0.941 (0.931, 0.949) | 0.908 (0.893, 0.922) | 0.854 (0.837, 0.871) | 0.978 (0.976, 0.980) | 0.880 (0.869, 0.893) | 0.897 (0.885, 0.910) | 0.869 (0.856, 0.882) |
|  | Macro average | 0.048 (0.044, 0.053) | 0.012 (0.011, 0.013) | 0.994 (0.993, 0.996) | 0.953 (0.945, 0.961) | 0.912 (0.901, 0.922) | 0.913 (0.901, 0.923) | 0.984 (0.983, 0.985) | 0.912 (0.903, 0.921) | 0.912 (0.902, 0.921) | 0.899 (0.890, 0.909) |
| BiomedBERT (AUROC; No, 3E-5, 256, 0.20, 0.005) | Original study | 0.057 (0.050, 0.064) | 0.013 (0.012, 0.015) | 0.997 (0.996, 0.998) | 0.998 (0.997, 0.999) | 0.988 (0.985, 0.990) | 0.987 (0.985, 0.989) | 0.984 (0.982, 0.986) | 0.987 (0.986, 0.989) | 0.987 (0.986, 0.989) | 0.964 (0.960, 0.968) |
|  | Review | 0.056 (0.050, 0.063) | 0.015 (0.013, 0.016) | 0.996 (0.995, 0.997) | 0.991 (0.989, 0.993) | 0.960 (0.954, 0.966) | 0.962 (0.956, 0.968) | 0.981 (0.979, 0.983) | 0.961 (0.957, 0.965) | 0.960 (0.955, 0.965) | 0.948 (0.942, 0.954) |
|  | Evidence-based guideline | 0.020 (0.017, 0.024) | 0.005 (0.005, 0.006) | 0.994 (0.991, 0.997) | 0.894 (0.866, 0.919) | 0.796 (0.752, 0.837) | 0.849 (0.805, 0.889) | 0.993 (0.992, 0.994) | 0.821 (0.784, 0.853) | 0.806 (0.765, 0.843) | 0.818 (0.780, 0.850) |
|  | Non-experimental | 0.064 (0.058, 0.070) | 0.018 (0.016, 0.019) | 0.991 (0.988, 0.993) | 0.933 (0.923, 0.943) | 0.875 (0.859, 0.892) | 0.862 (0.844, 0.878) | 0.977 (0.974, 0.979) | 0.869 (0.856, 0.881) | 0.873 (0.859, 0.887) | 0.856 (0.841, 0.869) |
|  | Macro average | 0.049 (0.045, 0.054) | 0.013 (0.012, 0.014) | 0.995 (0.993, 0.996) | 0.954 (0.946, 0.961) | 0.905 (0.893, 0.916) | 0.915 (0.903, 0.926) | 0.984 (0.982, 0.985) | 0.910 (0.899, 0.919) | 0.907 (0.896, 0.917) | 0.897 (0.885, 0.906) |
| BiomedBERT (AUPRC; No, 1E-5, 128, 0.05, 0.010) | Original study | 0.058 (0.051, 0.066) | 0.014 (0.012, 0.015) | 0.997 (0.996, 0.997) | 0.998 (0.997, 0.998) | 0.988 (0.986, 0.990) | 0.986 (0.984, 0.988) | 0.983 (0.981, 0.985) | 0.987 (0.986, 0.989) | 0.988 (0.986, 0.989) | 0.963 (0.959, 0.968) |
|  | Review | 0.057 (0.051, 0.063) | 0.015 (0.013, 0.016) | 0.996 (0.995, 0.997) | 0.991 (0.989, 0.993) | 0.967 (0.961, 0.972) | 0.956 (0.949, 0.962) | 0.981 (0.979, 0.983) | 0.961 (0.957, 0.966) | 0.964 (0.960, 0.969) | 0.949 (0.943, 0.954) |
|  | Evidence-based guideline | 0.021 (0.017, 0.024) | 0.005 (0.004, 0.006) | 0.994 (0.990, 0.997) | 0.890 (0.858, 0.917) | 0.799 (0.752, 0.842) | 0.855 (0.815, 0.894) | 0.993 (0.992, 0.995) | 0.826 (0.792, 0.858) | 0.809 (0.770, 0.847) | 0.823 (0.789, 0.856) |
|  | Non-experimental | 0.064 (0.058, 0.071) | 0.018 (0.016, 0.019) | 0.991 (0.989, 0.993) | 0.935 (0.925, 0.944) | 0.854 (0.836, 0.872) | 0.883 (0.866, 0.900) | 0.977 (0.975, 0.979) | 0.868 (0.855, 0.881) | 0.860 (0.844, 0.875) | 0.856 (0.841, 0.870) |
|  | Macro average | 0.050 (0.046, 0.054) | 0.013 (0.012, 0.014) | 0.995 (0.993, 0.996) | 0.953 (0.945, 0.961) | 0.902 (0.889, 0.914) | 0.920 (0.908, 0.931) | 0.984 (0.982, 0.985) | 0.911 (0.901, 0.920) | 0.905 (0.894, 0.916) | 0.898 (0.888, 0.908) |
| SciBERT-uncased (Recall; Yes, 3E-5, 256, 0.05, 0.010) | Original study | 0.071 (0.064, 0.079) | 0.017 (0.015, 0.019) | 0.997 (0.996, 0.998) | 0.998 (0.997, 0.999) | 0.974 (0.971, 0.977) | 0.994 (0.993, 0.996) | 0.979 (0.977, 0.982) | 0.984 (0.982, 0.986) | 0.978 (0.975, 0.980) | 0.956 (0.951, 0.961) |
|  | Review | 0.068 (0.061, 0.074) | 0.018 (0.017, 0.020) | 0.996 (0.995, 0.997) | 0.989 (0.987, 0.991) | 0.936 (0.929, 0.943) | 0.970 (0.964, 0.975) | 0.977 (0.975, 0.979) | 0.952 (0.948, 0.957) | 0.942 (0.936, 0.948) | 0.937 (0.931, 0.943) |
|  | Evidence-based guideline | 0.027 (0.023, 0.031) | 0.007 (0.006, 0.008) | 0.997 (0.995, 0.998) | 0.891 (0.862, 0.917) | 0.909 (0.875, 0.940) | 0.690 (0.646, 0.732) | 0.990 (0.989, 0.992) | 0.784 (0.751, 0.814) | 0.855 (0.824, 0.881) | 0.787 (0.755, 0.816) |
|  | Non-experimental | 0.083 (0.076, 0.089) | 0.023 (0.021, 0.025) | 0.988 (0.985, 0.990) | 0.926 (0.916, 0.936) | 0.918 (0.905, 0.932) | 0.779 (0.759, 0.799) | 0.970 (0.967, 0.972) | 0.843 (0.829, 0.856) | 0.887 (0.875, 0.899) | 0.830 (0.816, 0.844) |
|  | Macro average | 0.062 (0.058, 0.067) | 0.016 (0.015, 0.017) | 0.994 (0.993, 0.995) | 0.951 (0.943, 0.959) | 0.934 (0.925, 0.943) | 0.858 (0.847, 0.870) | 0.979 (0.978, 0.981) | 0.891 (0.881, 0.900) | 0.915 (0.906, 0.923) | 0.878 (0.867, 0.887) |
| BioLinkBERT (Precision; No, 3E-5, 16, 0.10, 0.010) | Original study | 0.068 (0.060, 0.077) | 0.014 (0.013, 0.016) | 0.995 (0.994, 0.996) | 0.997 (0.996, 0.998) | 0.988 (0.986, 0.990) | 0.987 (0.985, 0.989) | 0.984 (0.982, 0.986) | 0.987 (0.986, 0.989) | 0.988 (0.986, 0.989) | 0.965 (0.960, 0.969) |
|  | Review | 0.066 (0.059, 0.074) | 0.016 (0.015, 0.018) | 0.996 (0.995, 0.997) | 0.990 (0.988, 0.992) | 0.962 (0.956, 0.968) | 0.956 (0.949, 0.962) | 0.980 (0.977, 0.982) | 0.959 (0.954, 0.963) | 0.961 (0.956, 0.965) | 0.945 (0.939, 0.951) |
|  | Evidence-based guideline | 0.032 (0.026, 0.038) | 0.007 (0.005, 0.008) | 0.991 (0.987, 0.995) | 0.863 (0.830, 0.893) | 0.752 (0.703, 0.795) | 0.866 (0.825, 0.905) | 0.993 (0.992, 0.994) | 0.805 (0.768, 0.836) | 0.772 (0.729, 0.809) | 0.803 (0.767, 0.834) |
|  | Non-experimental | 0.081 (0.073, 0.090) | 0.020 (0.018, 0.022) | 0.990 (0.987, 0.992) | 0.920 (0.905, 0.934) | 0.872 (0.854, 0.889) | 0.869 (0.849, 0.885) | 0.977 (0.975, 0.979) | 0.870 (0.857, 0.882) | 0.871 (0.857, 0.886) | 0.858 (0.843, 0.871) |
|  | Macro average | 0.062 (0.056, 0.067) | 0.014 (0.013, 0.016) | 0.993 (0.992, 0.994) | 0.942 (0.933, 0.952) | 0.893 (0.880, 0.905) | 0.919 (0.908, 0.930) | 0.983 (0.982, 0.985) | 0.905 (0.894, 0.914) | 0.898 (0.886, 0.908) | 0.893 (0.881, 0.902) |
| BioBERT (Accuracy; No, 1E-5, 256, 0.05, 0.015) | Original study | 0.056 (0.050, 0.063) | 0.013 (0.012, 0.015) | 0.997 (0.996, 0.997) | 0.998 (0.997, 0.999) | 0.985 (0.983, 0.987) | 0.989 (0.987, 0.991) | 0.983 (0.981, 0.985) | 0.987 (0.985, 0.988) | 0.986 (0.984, 0.987) | 0.963 (0.959, 0.967) |
|  | Review | 0.055 (0.049, 0.061) | 0.014 (0.013, 0.016) | 0.996 (0.995, 0.997) | 0.990 (0.988, 0.993) | 0.963 (0.957, 0.969) | 0.962 (0.956, 0.968) | 0.982 (0.980, 0.984) | 0.963 (0.959, 0.967) | 0.963 (0.958, 0.968) | 0.950 (0.945, 0.956) |
|  | Evidence-based guideline | 0.020 (0.016, 0.023) | 0.005 (0.004, 0.006) | 0.994 (0.991, 0.997) | 0.889 (0.855, 0.917) | 0.799 (0.751, 0.844) | 0.852 (0.810, 0.890) | 0.993 (0.992, 0.995) | 0.825 (0.789, 0.857) | 0.809 (0.767, 0.847) | 0.822 (0.786, 0.854) |
|  | Non-experimental | 0.062 (0.057, 0.068) | 0.017 (0.016, 0.019) | 0.990 (0.987, 0.992) | 0.937 (0.927, 0.947) | 0.890 (0.873, 0.905) | 0.858 (0.840, 0.876) | 0.977 (0.975, 0.979) | 0.873 (0.861, 0.886) | 0.883 (0.868, 0.896) | 0.861 (0.848, 0.875) |
|  | Macro average | 0.048 (0.045, 0.052) | 0.013 (0.012, 0.014) | 0.994 (0.993, 0.995) | 0.954 (0.944, 0.961) | 0.909 (0.896, 0.921) | 0.915 (0.904, 0.925) | 0.984 (0.982, 0.985) | 0.912 (0.901, 0.921) | 0.910 (0.899, 0.921) | 0.899 (0.888, 0.909) |
| BioBERT (F1; No, 5E-5, 64, 0.10, 0.015) | Original study | 0.058 (0.051, 0.065) | 0.014 (0.012, 0.016) | 0.997 (0.996, 0.998) | 0.998 (0.997, 0.999) | 0.985 (0.983, 0.987) | 0.988 (0.986, 0.990) | 0.982 (0.980, 0.984) | 0.986 (0.985, 0.988) | 0.985 (0.984, 0.987) | 0.962 (0.957, 0.966) |
|  | Review | 0.061 (0.054, 0.068) | 0.015 (0.014, 0.017) | 0.996 (0.995, 0.997) | 0.990 (0.988, 0.992) | 0.957 (0.950, 0.963) | 0.967 (0.962, 0.973) | 0.981 (0.979, 0.983) | 0.962 (0.958, 0.966) | 0.959 (0.953, 0.964) | 0.950 (0.944, 0.955) |
|  | Evidence-based guideline | 0.024 (0.020, 0.028) | 0.006 (0.005, 0.007) | 0.994 (0.990, 0.997) | 0.878 (0.846, 0.904) | 0.792 (0.746, 0.833) | 0.826 (0.786, 0.869) | 0.993 (0.991, 0.994) | 0.809 (0.775, 0.839) | 0.799 (0.759, 0.832) | 0.805 (0.771, 0.836) |
|  | Non-experimental | 0.063 (0.058, 0.069) | 0.018 (0.016, 0.019) | 0.992 (0.989, 0.993) | 0.935 (0.925, 0.945) | 0.899 (0.885, 0.913) | 0.846 (0.827, 0.863) | 0.977 (0.974, 0.979) | 0.872 (0.859, 0.883) | 0.888 (0.875, 0.900) | 0.859 (0.846, 0.872) |
|  | Macro average | 0.051 (0.048, 0.056) | 0.013 (0.012, 0.014) | 0.995 (0.993, 0.996) | 0.951 (0.942, 0.958) | 0.908 (0.896, 0.919) | 0.907 (0.896, 0.919) | 0.983 (0.982, 0.985) | 0.907 (0.897, 0.917) | 0.908 (0.897, 0.918) | 0.894 (0.883, 0.904) |
| BiomedBERT (F2; Yes, 3E-5, 128, 0.05, 0.015) | Original study | 0.063 (0.056, 0.072) | 0.014 (0.013, 0.016) | 0.996 (0.995, 0.997) | 0.998 (0.997, 0.998) | 0.982 (0.979, 0.984) | 0.992 (0.990, 0.994) | 0.983 (0.981, 0.985) | 0.987 (0.985, 0.988) | 0.984 (0.982, 0.986) | 0.963 (0.959, 0.968) |
|  | Review | 0.060 (0.054, 0.067) | 0.015 (0.014, 0.017) | 0.996 (0.995, 0.997) | 0.987 (0.984, 0.990) | 0.951 (0.944, 0.958) | 0.971 (0.965, 0.976) | 0.981 (0.979, 0.983) | 0.961 (0.956, 0.965) | 0.955 (0.949, 0.961) | 0.948 (0.942, 0.954) |
|  | Evidence-based guideline | 0.028 (0.023, 0.034) | 0.006 (0.005, 0.007) | 0.992 (0.987, 0.995) | 0.874 (0.840, 0.906) | 0.855 (0.816, 0.893) | 0.775 (0.733, 0.817) | 0.992 (0.991, 0.994) | 0.813 (0.780, 0.844) | 0.838 (0.803, 0.869) | 0.810 (0.777, 0.841) |
|  | Non-experimental | 0.071 (0.065, 0.077) | 0.019 (0.018, 0.021) | 0.991 (0.989, 0.992) | 0.928 (0.915, 0.938) | 0.913 (0.898, 0.926) | 0.822 (0.804, 0.842) | 0.975 (0.972, 0.977) | 0.865 (0.853, 0.877) | 0.893 (0.881, 0.905) | 0.853 (0.839, 0.866) |
|  | Macro average | 0.055 (0.051, 0.060) | 0.014 (0.013, 0.015) | 0.994 (0.992, 0.995) | 0.947 (0.937, 0.955) | 0.925 (0.915, 0.936) | 0.890 (0.877, 0.901) | 0.983 (0.981, 0.984) | 0.907 (0.896, 0.916) | 0.917 (0.907, 0.927) | 0.894 (0.882, 0.904) |
| BiomedBERT (MCC; No, 1E-5, 16, 0.20, 0.005) | Original study | 0.073 (0.064, 0.082) | 0.015 (0.013, 0.016) | 0.996 (0.995, 0.997) | 0.998 (0.997, 0.998) | 0.986 (0.983, 0.988) | 0.989 (0.986, 0.991) | 0.983 (0.981, 0.985) | 0.987 (0.986, 0.989) | 0.986 (0.984, 0.988) | 0.964 (0.959, 0.968) |
|  | Review | 0.070 (0.062, 0.078) | 0.017 (0.015, 0.018) | 0.996 (0.995, 0.997) | 0.990 (0.987, 0.992) | 0.959 (0.952, 0.965) | 0.958 (0.952, 0.964) | 0.979 (0.977, 0.982) | 0.958 (0.954, 0.963) | 0.958 (0.953, 0.964) | 0.945 (0.939, 0.951) |
|  | Evidence-based guideline | 0.028 (0.023, 0.035) | 0.006 (0.005, 0.007) | 0.992 (0.987, 0.996) | 0.870 (0.836, 0.899) | 0.767 (0.718, 0.813) | 0.838 (0.794, 0.881) | 0.993 (0.991, 0.994) | 0.801 (0.763, 0.836) | 0.781 (0.737, 0.819) | 0.798 (0.761, 0.833) |
|  | Non-experimental | 0.077 (0.069, 0.085) | 0.019 (0.018, 0.021) | 0.989 (0.986, 0.992) | 0.929 (0.918, 0.940) | 0.884 (0.866, 0.901) | 0.852 (0.834, 0.870) | 0.976 (0.974, 0.979) | 0.868 (0.854, 0.880) | 0.878 (0.862, 0.891) | 0.855 (0.840, 0.868) |
|  | Macro average | 0.062 (0.056, 0.067) | 0.014 (0.013, 0.015) | 0.993 (0.992, 0.995) | 0.947 (0.937, 0.955) | 0.899 (0.886, 0.911) | 0.909 (0.897, 0.921) | 0.983 (0.982, 0.984) | 0.904 (0.893, 0.914) | 0.901 (0.889, 0.912) | 0.890 (0.879, 0.902) |

**AP** Average precision; **AUROC** Area under the receiver operating characteristic curve; **BS** Batch size; **CW** Class weights; **LR** Learning rate; **MCC** Matthews correlation coefficient; **WD** Weight decay; **WR** Warmup ratio.

**Note:** All values are presented as score (95% CI).

# **Table S6. Performance of the top models on Clinical Hedges**

| **Model (Best metric; CW, LR, BS, WR, WD)** | **Class** | **Cross-entropy Loss** | **Brier Score** | **AUROC** | **AUPRC** | **Recall** | **Precision** | **Accuracy** | **F1 Score** | **F2 Score** | **MCC** |
| --- | --- | --- | --- | --- | --- | --- | --- | --- | --- | --- | --- |
| BioBERT (Cross entropy loss; No, 5E-5, 256, 0.10, 0.015) | Original study | 0.271 (0.263, 0.279) | 0.076 (0.074, 0.078) | 0.974 (0.973, 0.975) | 0.981 (0.979, 0.982) | 0.841 (0.837, 0.845) | 0.974 (0.972, 0.976) | 0.903 (0.900, 0.905) | 0.903 (0.900, 0.906) | 0.865 (0.861, 0.869) | 0.816 (0.811, 0.820) |
|  | Review | 0.113 (0.108, 0.118) | 0.029 (0.028, 0.031) | 0.944 (0.940, 0.948) | 0.750 (0.736, 0.763) | 0.644 (0.628, 0.662) | 0.732 (0.716, 0.747) | 0.962 (0.961, 0.964) | 0.685 (0.671, 0.698) | 0.660 (0.645, 0.675) | 0.667 (0.652, 0.680) |
|  | Evidence-based guideline | 0.296 (0.290, 0.304) | 0.090 (0.088, 0.092) | 0.958 (0.957, 0.960) | 0.926 (0.922, 0.930) | 0.942 (0.939, 0.946) | 0.791 (0.785, 0.796) | 0.877 (0.874, 0.880) | 0.860 (0.856, 0.864) | 0.908 (0.905, 0.911) | 0.761 (0.756, 0.767) |
|  | Macro average | 0.227 (0.222, 0.233) | 0.065 (0.064, 0.067) | 0.959 (0.957, 0.961) | 0.885 (0.880, 0.891) | 0.809 (0.803, 0.815) | 0.832 (0.826, 0.838) | 0.914 (0.912, 0.916) | 0.816 (0.810, 0.821) | 0.811 (0.805, 0.816) | 0.748 (0.741, 0.755) |
| BioBERT (Brier score; No, 1E-5, 64, 0.20, 0.015) | Original study | 0.227 (0.219, 0.235) | 0.056 (0.054, 0.058) | 0.975 (0.974, 0.977) | 0.982 (0.981, 0.983) | 0.886 (0.882, 0.890) | 0.976 (0.974, 0.978) | 0.927 (0.925, 0.930) | 0.929 (0.927, 0.931) | 0.903 (0.899, 0.906) | 0.859 (0.855, 0.864) |
|  | Review | 0.109 (0.104, 0.114) | 0.028 (0.027, 0.029) | 0.951 (0.947, 0.955) | 0.766 (0.753, 0.779) | 0.634 (0.618, 0.651) | 0.767 (0.751, 0.784) | 0.964 (0.963, 0.966) | 0.695 (0.681, 0.709) | 0.657 (0.642, 0.673) | 0.679 (0.665, 0.694) |
|  | Evidence-based guideline | 0.263 (0.256, 0.270) | 0.074 (0.072, 0.076) | 0.964 (0.963, 0.966) | 0.931 (0.927, 0.934) | 0.947 (0.944, 0.950) | 0.830 (0.825, 0.835) | 0.901 (0.898, 0.904) | 0.884 (0.881, 0.888) | 0.921 (0.918, 0.924) | 0.804 (0.798, 0.809) |
|  | Macro average | 0.200 (0.195, 0.205) | 0.053 (0.051, 0.054) | 0.964 (0.962, 0.965) | 0.893 (0.888, 0.898) | 0.822 (0.817, 0.828) | 0.858 (0.852, 0.864) | 0.931 (0.929, 0.933) | 0.836 (0.831, 0.842) | 0.827 (0.821, 0.833) | 0.781 (0.774, 0.787) |
| BiomedBERT (AUROC; No, 3E-5, 256, 0.20, 0.005) | Original study | 0.233 (0.225, 0.241) | 0.058 (0.056, 0.060) | 0.974 (0.973, 0.976) | 0.981 (0.980, 0.982) | 0.889 (0.885, 0.892) | 0.970 (0.968, 0.972) | 0.926 (0.923, 0.928) | 0.928 (0.925, 0.930) | 0.904 (0.901, 0.907) | 0.855 (0.850, 0.860) |
|  | Review | 0.115 (0.110, 0.120) | 0.031 (0.030, 0.033) | 0.953 (0.949, 0.957) | 0.762 (0.749, 0.775) | 0.681 (0.664, 0.698) | 0.673 (0.657, 0.689) | 0.959 (0.957, 0.960) | 0.677 (0.664, 0.690) | 0.679 (0.665, 0.694) | 0.655 (0.641, 0.669) |
|  | Evidence-based guideline | 0.272 (0.265, 0.279) | 0.079 (0.077, 0.081) | 0.961 (0.959, 0.962) | 0.927 (0.923, 0.931) | 0.917 (0.913, 0.921) | 0.835 (0.830, 0.840) | 0.894 (0.891, 0.897) | 0.874 (0.871, 0.878) | 0.899 (0.896, 0.903) | 0.785 (0.780, 0.791) |
|  | Macro average | 0.207 (0.201, 0.212) | 0.056 (0.055, 0.057) | 0.963 (0.961, 0.964) | 0.890 (0.885, 0.895) | 0.829 (0.823, 0.835) | 0.826 (0.820, 0.832) | 0.926 (0.924, 0.928) | 0.826 (0.821, 0.831) | 0.828 (0.822, 0.833) | 0.765 (0.759, 0.771) |
| BiomedBERT (AUPRC; No, 1E-5, 128, 0.05, 0.010) | Original study | 0.213 (0.204, 0.220) | 0.049 (0.047, 0.051) | 0.975 (0.974, 0.976) | 0.982 (0.981, 0.983) | 0.926 (0.922, 0.929) | 0.965 (0.963, 0.968) | 0.942 (0.940, 0.945) | 0.945 (0.943, 0.947) | 0.933 (0.930, 0.936) | 0.885 (0.881, 0.890) |
|  | Review | 0.111 (0.106, 0.116) | 0.030 (0.029, 0.032) | 0.955 (0.952, 0.959) | 0.772 (0.758, 0.785) | 0.693 (0.676, 0.709) | 0.681 (0.664, 0.697) | 0.960 (0.958, 0.962) | 0.687 (0.673, 0.700) | 0.690 (0.675, 0.705) | 0.665 (0.651, 0.679) |
|  | Evidence-based guideline | 0.253 (0.246, 0.261) | 0.070 (0.068, 0.072) | 0.965 (0.963, 0.966) | 0.932 (0.928, 0.935) | 0.911 (0.907, 0.915) | 0.875 (0.870, 0.879) | 0.912 (0.909, 0.915) | 0.893 (0.889, 0.896) | 0.904 (0.900, 0.907) | 0.819 (0.813, 0.824) |
|  | Macro average | 0.192 (0.187, 0.198) | 0.050 (0.049, 0.051) | 0.965 (0.963, 0.967) | 0.895 (0.890, 0.900) | 0.843 (0.837, 0.849) | 0.840 (0.834, 0.846) | 0.938 (0.936, 0.940) | 0.842 (0.836, 0.847) | 0.843 (0.837, 0.848) | 0.790 (0.783, 0.796) |
| SciBERT-uncased (Recall; Yes, 3E-5, 256, 0.05, 0.010) | Original study | 0.369 (0.359, 0.380) | 0.093 (0.090, 0.095) | 0.975 (0.973, 0.976) | 0.981 (0.980, 0.982) | 0.802 (0.797, 0.807) | 0.987 (0.986, 0.989) | 0.888 (0.885, 0.891) | 0.885 (0.882, 0.888) | 0.833 (0.829, 0.837) | 0.795 (0.790, 0.799) |
|  | Review | 0.130 (0.124, 0.136) | 0.030 (0.029, 0.032) | 0.939 (0.935, 0.944) | 0.738 (0.724, 0.752) | 0.538 (0.520, 0.555) | 0.837 (0.820, 0.853) | 0.964 (0.962, 0.966) | 0.655 (0.639, 0.671) | 0.580 (0.562, 0.597) | 0.654 (0.639, 0.670) |
|  | Evidence-based guideline | 0.416 (0.406, 0.426) | 0.112 (0.109, 0.114) | 0.959 (0.957, 0.961) | 0.926 (0.923, 0.930) | 0.971 (0.969, 0.974) | 0.755 (0.749, 0.760) | 0.862 (0.859, 0.865) | 0.850 (0.846, 0.853) | 0.919 (0.916, 0.921) | 0.746 (0.740, 0.751) |
|  | Macro average | 0.305 (0.298, 0.312) | 0.078 (0.077, 0.080) | 0.958 (0.956, 0.960) | 0.882 (0.876, 0.887) | 0.771 (0.764, 0.777) | 0.859 (0.854, 0.866) | 0.905 (0.903, 0.907) | 0.797 (0.790, 0.803) | 0.777 (0.771, 0.784) | 0.732 (0.725, 0.738) |
| BioLinkBERT (Precision; No, 3E-5, 16, 0.10, 0.010) | Original study | 0.373 (0.363, 0.385) | 0.078 (0.076, 0.081) | 0.961 (0.959, 0.963) | 0.973 (0.972, 0.975) | 0.865 (0.861, 0.869) | 0.973 (0.971, 0.975) | 0.915 (0.912, 0.917) | 0.916 (0.913, 0.918) | 0.885 (0.881, 0.888) | 0.836 (0.831, 0.840) |
|  | Review | 0.158 (0.150, 0.166) | 0.034 (0.033, 0.036) | 0.941 (0.936, 0.945) | 0.737 (0.723, 0.752) | 0.659 (0.642, 0.675) | 0.683 (0.667, 0.700) | 0.959 (0.957, 0.961) | 0.671 (0.658, 0.685) | 0.664 (0.650, 0.679) | 0.649 (0.635, 0.664) |
|  | Evidence-based guideline | 0.456 (0.444, 0.469) | 0.101 (0.099, 0.104) | 0.951 (0.949, 0.953) | 0.878 (0.871, 0.884) | 0.934 (0.931, 0.938) | 0.815 (0.809, 0.820) | 0.889 (0.886, 0.891) | 0.870 (0.867, 0.874) | 0.908 (0.904, 0.911) | 0.779 (0.773, 0.784) |
|  | Macro average | 0.329 (0.321, 0.338) | 0.071 (0.070, 0.073) | 0.951 (0.949, 0.953) | 0.863 (0.857, 0.869) | 0.820 (0.814, 0.825) | 0.824 (0.818, 0.829) | 0.921 (0.919, 0.923) | 0.819 (0.814, 0.824) | 0.819 (0.813, 0.824) | 0.755 (0.748, 0.761) |
| BioBERT (Accuracy; No, 1E-5, 256, 0.05, 0.015) | Original study | 0.211 (0.204, 0.218) | 0.052 (0.050, 0.053) | 0.976 (0.975, 0.978) | 0.983 (0.982, 0.984) | 0.909 (0.905, 0.912) | 0.977 (0.975, 0.979) | 0.940 (0.938, 0.942) | 0.942 (0.940, 0.944) | 0.922 (0.919, 0.925) | 0.882 (0.878, 0.886) |
|  | Review | 0.106 (0.102, 0.111) | 0.028 (0.027, 0.029) | 0.951 (0.948, 0.955) | 0.768 (0.755, 0.781) | 0.647 (0.630, 0.664) | 0.763 (0.747, 0.780) | 0.965 (0.963, 0.966) | 0.700 (0.687, 0.713) | 0.667 (0.652, 0.682) | 0.684 (0.671, 0.697) |
|  | Evidence-based guideline | 0.244 (0.238, 0.251) | 0.069 (0.067, 0.071) | 0.967 (0.965, 0.968) | 0.936 (0.932, 0.940) | 0.946 (0.943, 0.949) | 0.854 (0.850, 0.859) | 0.914 (0.911, 0.916) | 0.898 (0.895, 0.901) | 0.926 (0.923, 0.929) | 0.826 (0.822, 0.831) |
|  | Macro average | 0.187 (0.182, 0.192) | 0.049 (0.048, 0.051) | 0.965 (0.963, 0.966) | 0.896 (0.891, 0.901) | 0.834 (0.828, 0.840) | 0.865 (0.859, 0.870) | 0.939 (0.938, 0.941) | 0.846 (0.842, 0.851) | 0.838 (0.833, 0.844) | 0.798 (0.792, 0.803) |
| BioBERT (F1; No, 5E-5, 64, 0.10, 0.015) | Original study | 0.274 (0.265, 0.284) | 0.065 (0.063, 0.067) | 0.972 (0.970, 0.973) | 0.979 (0.977, 0.980) | 0.879 (0.875, 0.883) | 0.972 (0.970, 0.974) | 0.922 (0.919, 0.924) | 0.923 (0.921, 0.926) | 0.896 (0.893, 0.899) | 0.848 (0.843, 0.852) |
|  | Review | 0.133 (0.127, 0.140) | 0.034 (0.032, 0.035) | 0.948 (0.944, 0.952) | 0.737 (0.724, 0.751) | 0.630 (0.613, 0.646) | 0.682 (0.666, 0.699) | 0.958 (0.956, 0.959) | 0.655 (0.642, 0.669) | 0.640 (0.625, 0.655) | 0.633 (0.620, 0.648) |
|  | Evidence-based guideline | 0.325 (0.316, 0.334) | 0.088 (0.085, 0.090) | 0.958 (0.956, 0.960) | 0.920 (0.916, 0.924) | 0.928 (0.924, 0.932) | 0.821 (0.816, 0.826) | 0.890 (0.887, 0.893) | 0.871 (0.868, 0.875) | 0.904 (0.901, 0.908) | 0.781 (0.775, 0.786) |
|  | Macro average | 0.244 (0.238, 0.251) | 0.062 (0.060, 0.064) | 0.959 (0.957, 0.961) | 0.879 (0.874, 0.884) | 0.812 (0.806, 0.818) | 0.825 (0.819, 0.831) | 0.923 (0.921, 0.925) | 0.817 (0.812, 0.822) | 0.813 (0.808, 0.819) | 0.754 (0.748, 0.760) |
| BiomedBERT (F2; Yes, 3E-5, 128, 0.05, 0.015) | Original study | 0.329 (0.318, 0.339) | 0.076 (0.074, 0.078) | 0.974 (0.973, 0.976) | 0.981 (0.980, 0.982) | 0.845 (0.840, 0.849) | 0.983 (0.981, 0.984) | 0.909 (0.906, 0.911) | 0.908 (0.906, 0.911) | 0.869 (0.865, 0.873) | 0.828 (0.823, 0.832) |
|  | Review | 0.113 (0.108, 0.118) | 0.029 (0.028, 0.030) | 0.946 (0.942, 0.950) | 0.755 (0.741, 0.767) | 0.603 (0.586, 0.619) | 0.783 (0.765, 0.799) | 0.964 (0.962, 0.966) | 0.681 (0.667, 0.695) | 0.632 (0.616, 0.648) | 0.669 (0.655, 0.682) |
|  | Evidence-based guideline | 0.349 (0.341, 0.358) | 0.095 (0.093, 0.098) | 0.958 (0.956, 0.959) | 0.925 (0.922, 0.929) | 0.958 (0.955, 0.960) | 0.791 (0.786, 0.796) | 0.882 (0.879, 0.885) | 0.866 (0.863, 0.870) | 0.919 (0.916, 0.921) | 0.773 (0.768, 0.778) |
|  | Macro average | 0.264 (0.257, 0.270) | 0.067 (0.065, 0.068) | 0.959 (0.958, 0.961) | 0.887 (0.882, 0.892) | 0.802 (0.796, 0.808) | 0.852 (0.846, 0.858) | 0.918 (0.916, 0.920) | 0.819 (0.813, 0.824) | 0.807 (0.801, 0.812) | 0.757 (0.751, 0.763) |
| BiomedBERT (MCC; No, 1E-5, 16, 0.20, 0.005) | Original study | 0.365 (0.354, 0.377) | 0.079 (0.076, 0.081) | 0.967 (0.965, 0.968) | 0.976 (0.975, 0.977) | 0.859 (0.854, 0.863) | 0.975 (0.973, 0.977) | 0.913 (0.910, 0.915) | 0.913 (0.911, 0.916) | 0.880 (0.876, 0.884) | 0.833 (0.828, 0.838) |
|  | Review | 0.127 (0.121, 0.133) | 0.031 (0.030, 0.032) | 0.949 (0.945, 0.953) | 0.758 (0.745, 0.771) | 0.651 (0.634, 0.668) | 0.737 (0.721, 0.753) | 0.963 (0.961, 0.965) | 0.691 (0.677, 0.704) | 0.666 (0.651, 0.682) | 0.673 (0.658, 0.687) |
|  | Evidence-based guideline | 0.396 (0.386, 0.406) | 0.099 (0.096, 0.101) | 0.954 (0.953, 0.956) | 0.914 (0.910, 0.918) | 0.942 (0.939, 0.946) | 0.806 (0.800, 0.811) | 0.886 (0.883, 0.889) | 0.869 (0.865, 0.872) | 0.911 (0.908, 0.914) | 0.776 (0.771, 0.781) |
|  | Macro average | 0.296 (0.289, 0.304) | 0.070 (0.068, 0.071) | 0.957 (0.955, 0.959) | 0.883 (0.878, 0.888) | 0.817 (0.811, 0.823) | 0.839 (0.834, 0.845) | 0.921 (0.919, 0.922) | 0.824 (0.819, 0.830) | 0.819 (0.813, 0.825) | 0.761 (0.754, 0.767) |

**AP** Average precision; **AUROC** Area under the receiver operating characteristic curve; **BS** Batch size; **CW** Class weights; **LR** Learning rate; **MCC** Matthews correlation coefficient; **WD** Weight decay; **WR** Warmup ratio.

**Note:** All values are presented as score (95% CI).

# **Figure S1.** Aggregated model performance on PLUS-validate by pretrained model


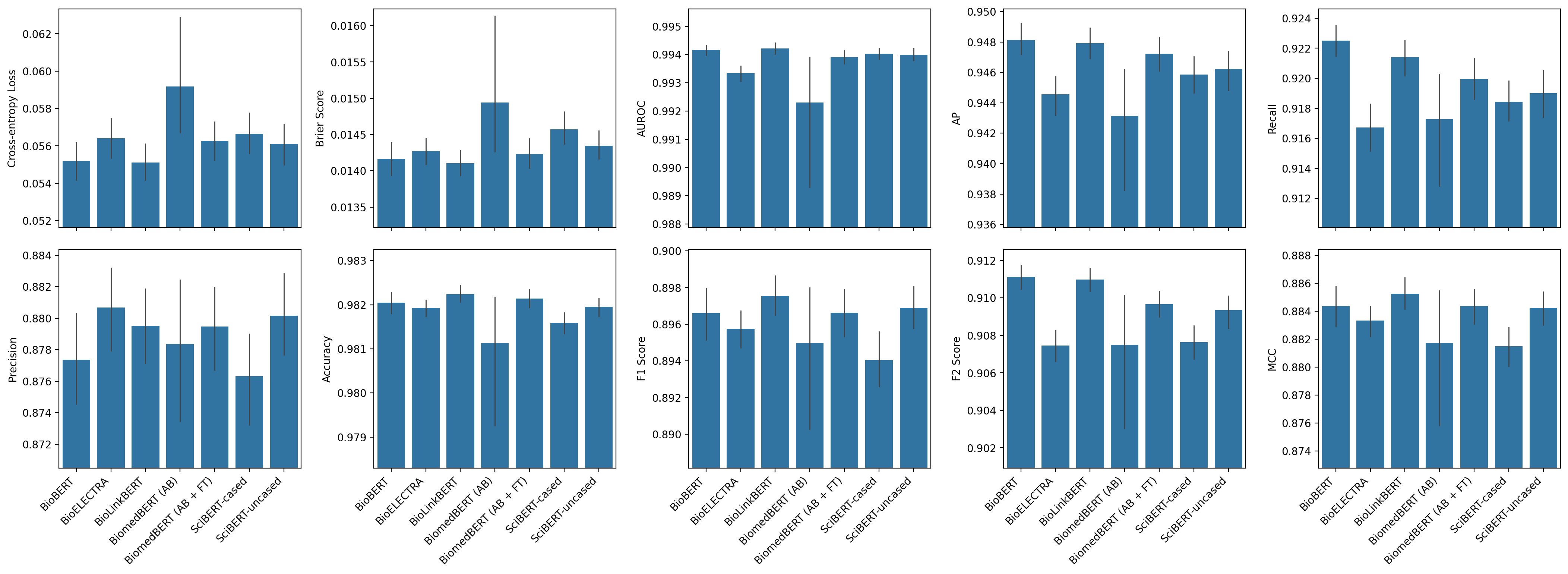


**Note:** Error bars represent 95% CI.

# **Figure S2.** Aggregated model performance on PLUS-validate by class weight adjustment


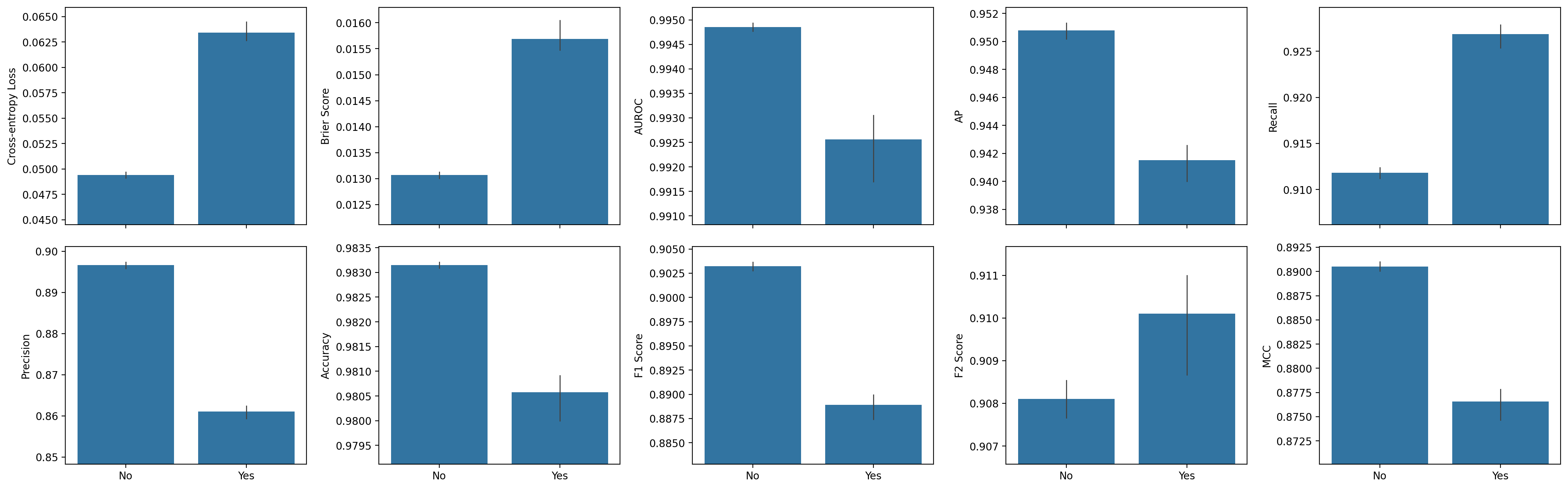


**Note:** Error bars represent 95% CI.

# **Figure S3.** Aggregated model performance on PLUS-validate by learning rate


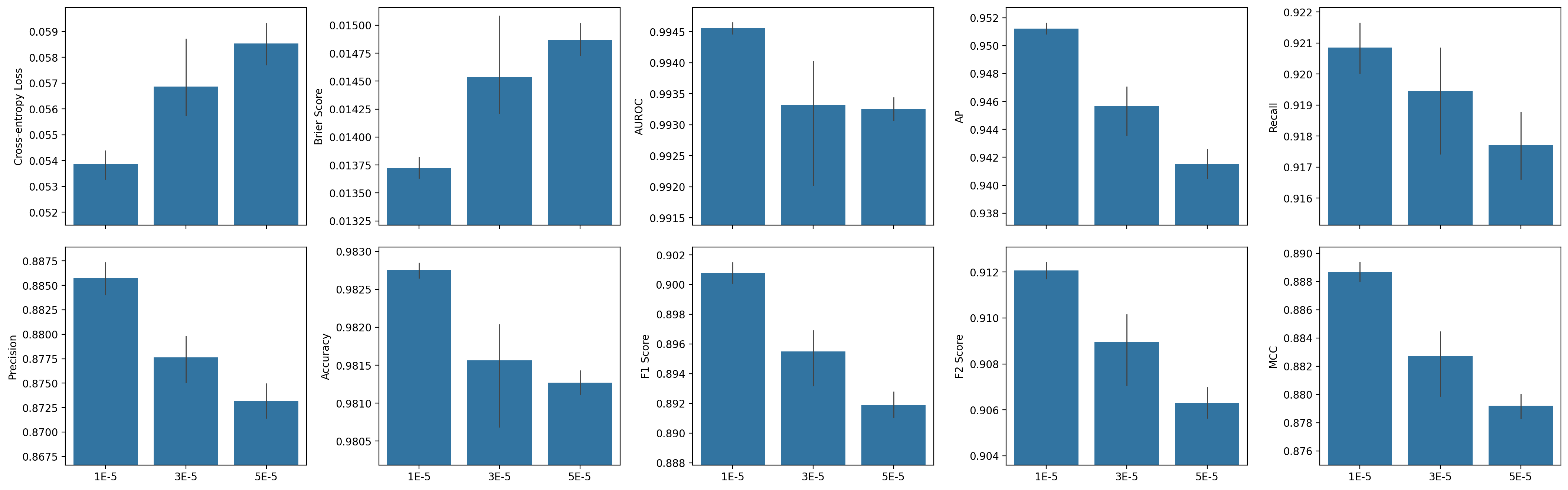


**Note:** Error bars represent 95% CI.

# **Figure S4.** Aggregated model performance on PLUS-validate by batch size

**
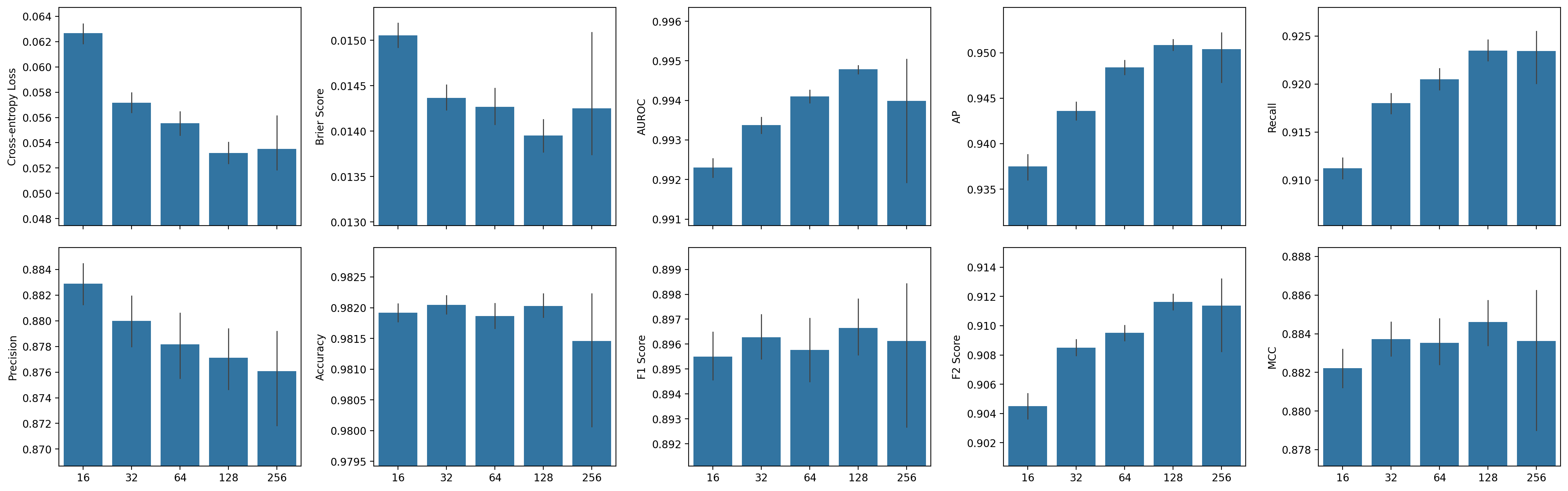
**

**Note:** Error bars represent 95% CI.

# **Figure S5.** Aggregated model performance on PLUS-validate by warmup ratio

**
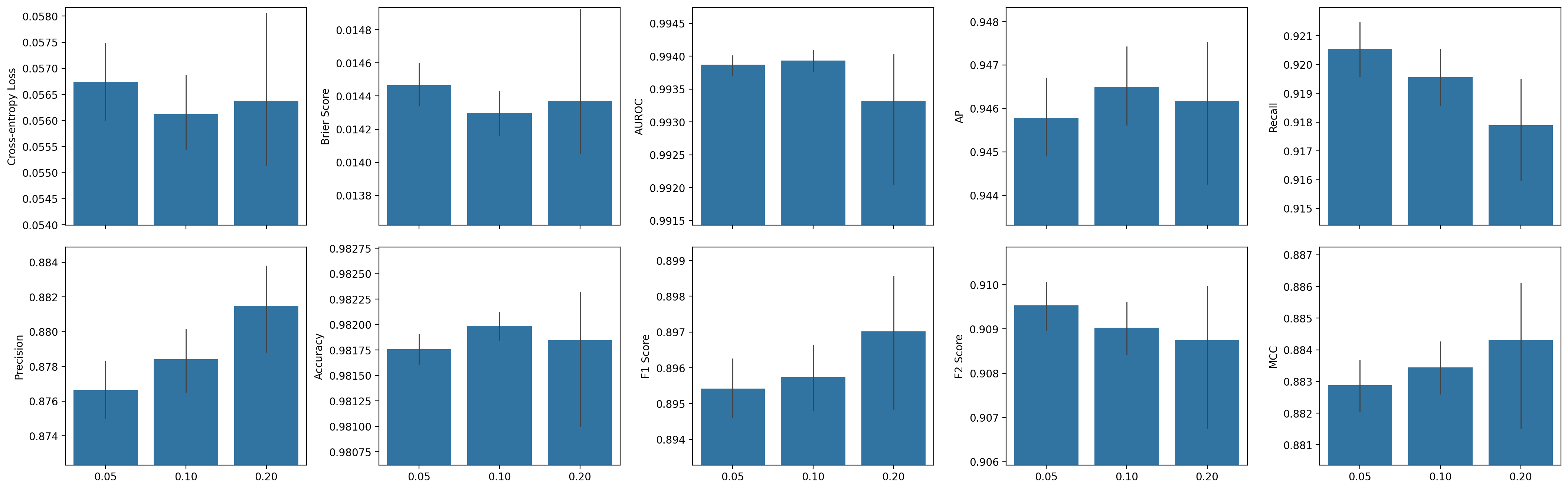
**

**Note:** Error bars represent 95% CI.

# **Figure S6.** Aggregated model performance on PLUS-validate by weight decay

**
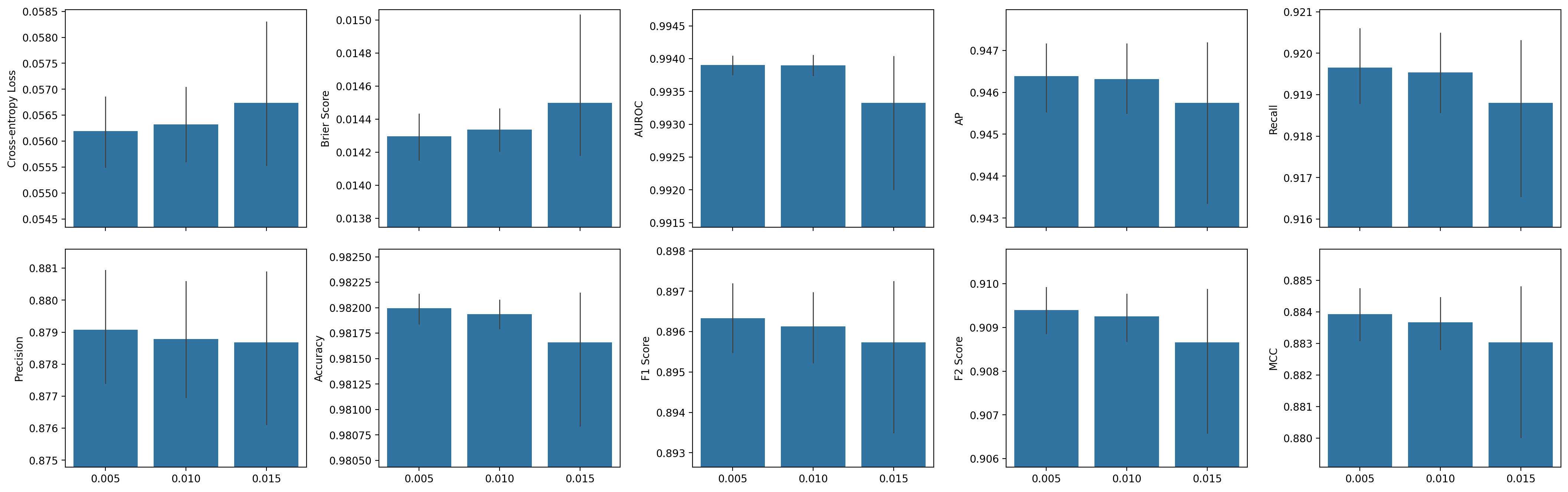
**

**Note:** Error bars represent 95% CI.

# **Figure S7.** Confusion matrices for the best-cross entropy loss model (BioBERT; CW: No; LR: 5E-5; BS: 256; WR: 0.10; WD: 0.015)


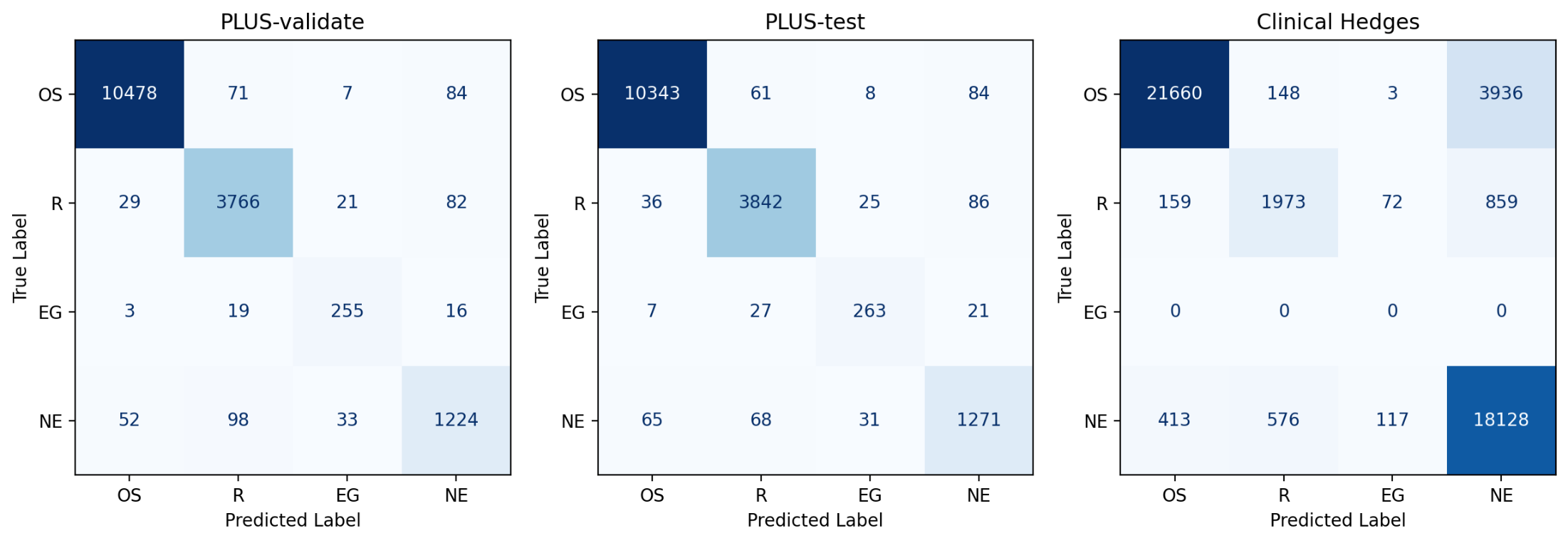


**EG** Evidence-based guideline; **NE** Non-experimental; **OS** Original study; **R** Review.

# **Figure S8.** Confusion matrices for the best-Brier score model (BioBERT; CW: No; LR: 1E-5; BS: 64; WR: 0.20; WD: 0.015)


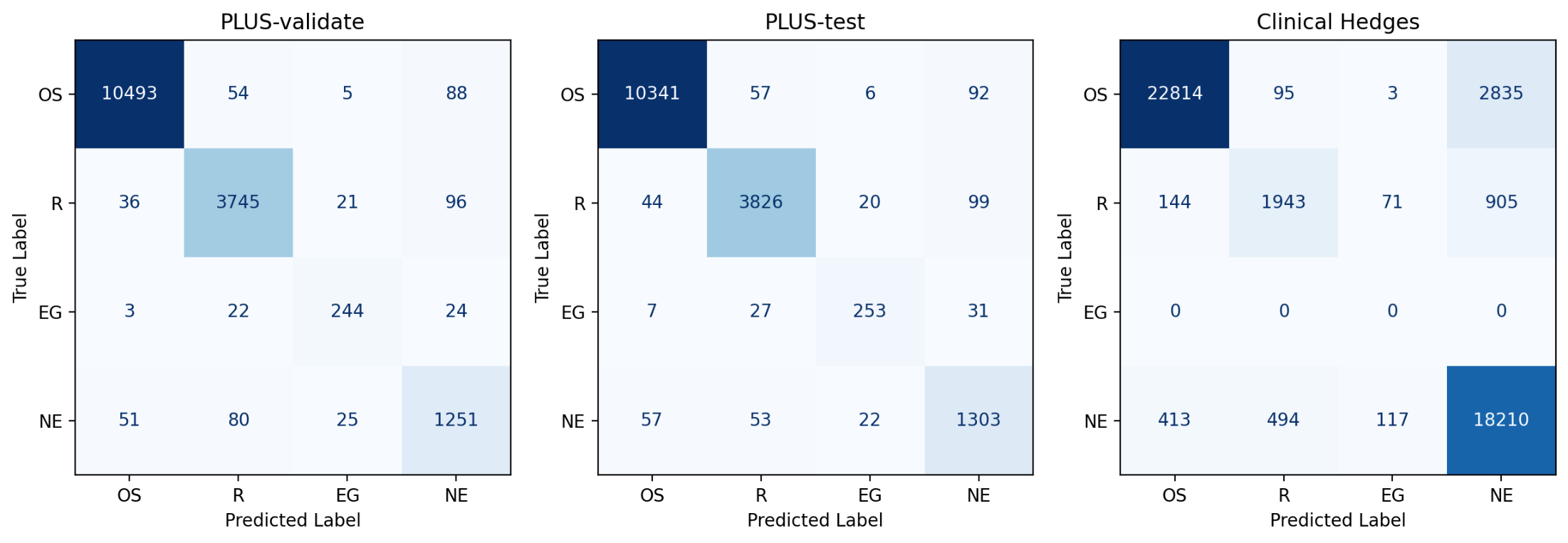


**EG** Evidence-based guideline; **NE** Non-experimental; **OS** Original study; **R** Review.

# **Figure S9.** Confusion matrices for the best-AP model (BiomedBERT; CW: No; LR: 1E-5; BS: 128; WR: 0.05; WD: 0.010)


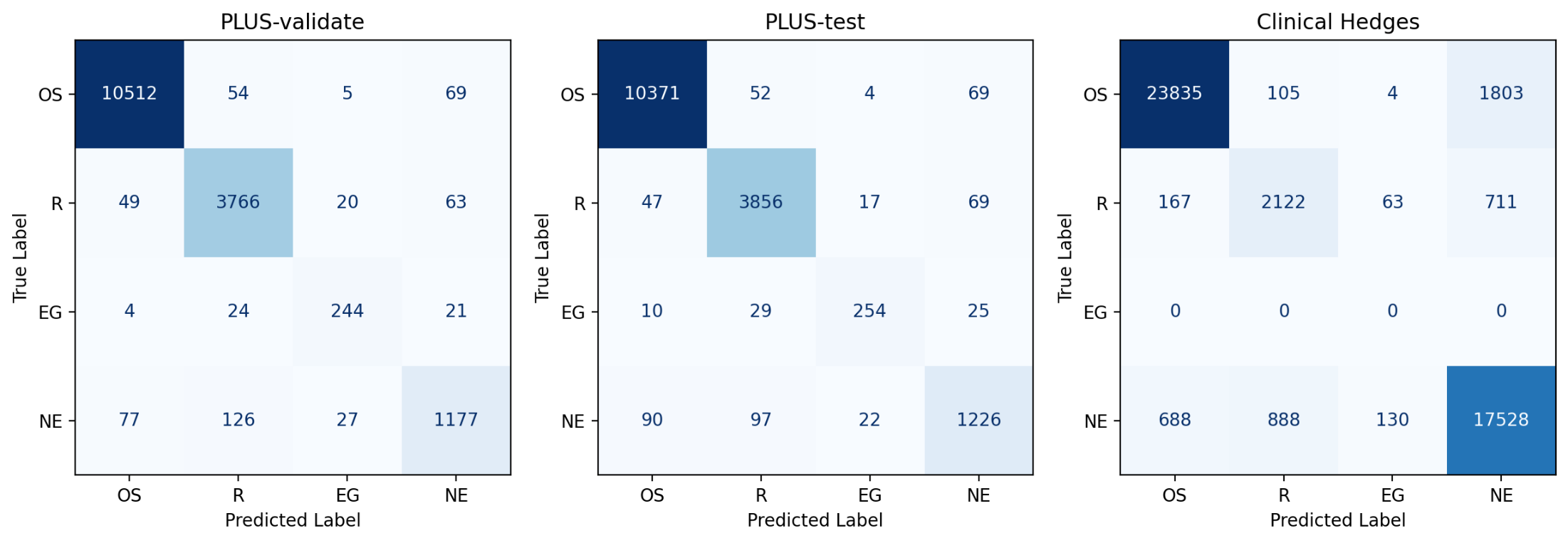


**EG** Evidence-based guideline; **NE** Non-experimental; **OS** Original study; **R** Review.

# **Figure S10.** Confusion matrices for the best-recall model (SciBERT-uncased; CW: Yes; LR: 3E-5; BS: 256; WR: 0.05; WD: 0.010)


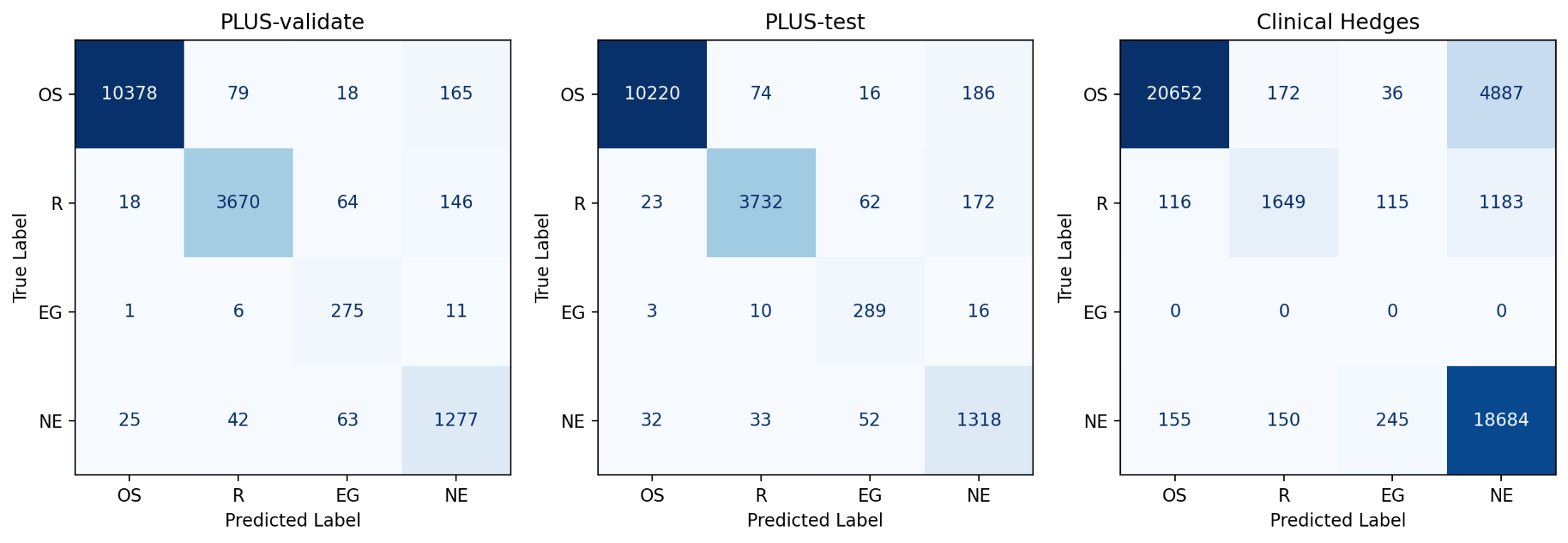


**EG** Evidence-based guideline; **NE** Non-experimental; **OS** Original study; **R** Review.

# **Figure S11.** Confusion matrices for the best-precision model (BioLinkBERT; CW: No; LR: 3E-5; BS: 16; WR: 0.10; WD: 0.010)

**
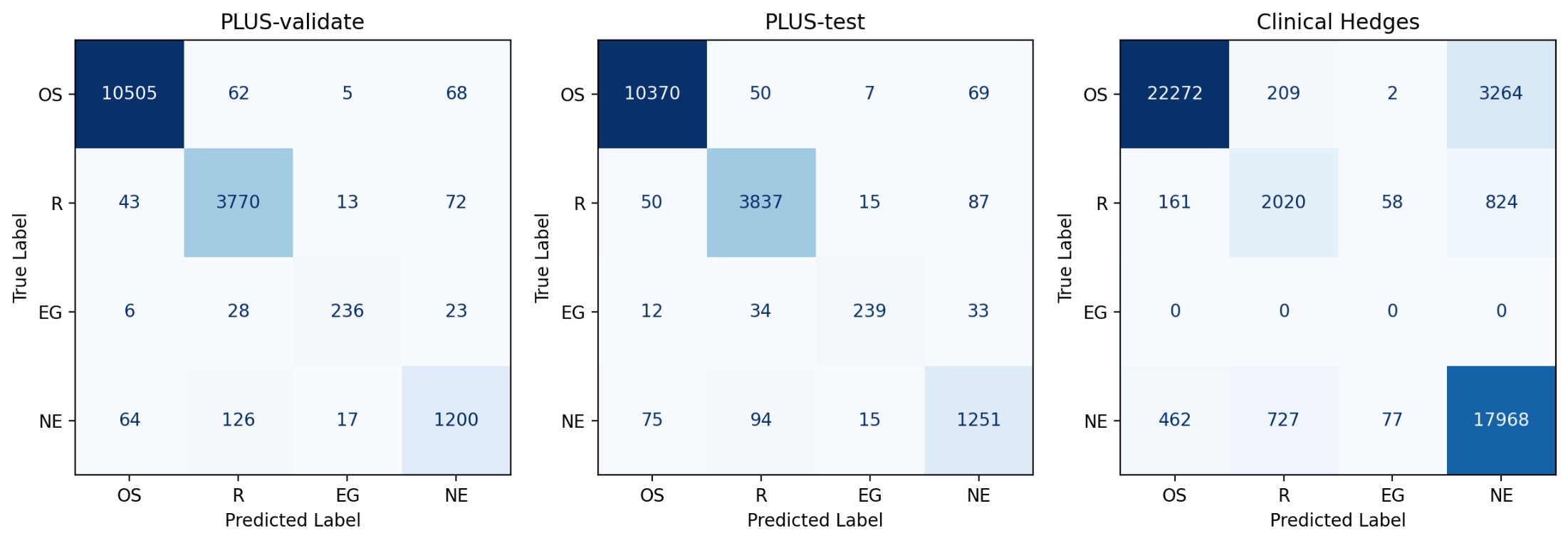
**

**EG** Evidence-based guideline; **NE** Non-experimental; **OS** Original study; **R** Review.

# **Figure S12.** Confusion matrices for the best-accuracy model (BioBERT; CW: No; LR: 1E-5; BS: 256; WR: 0.05; WD: 0.015)


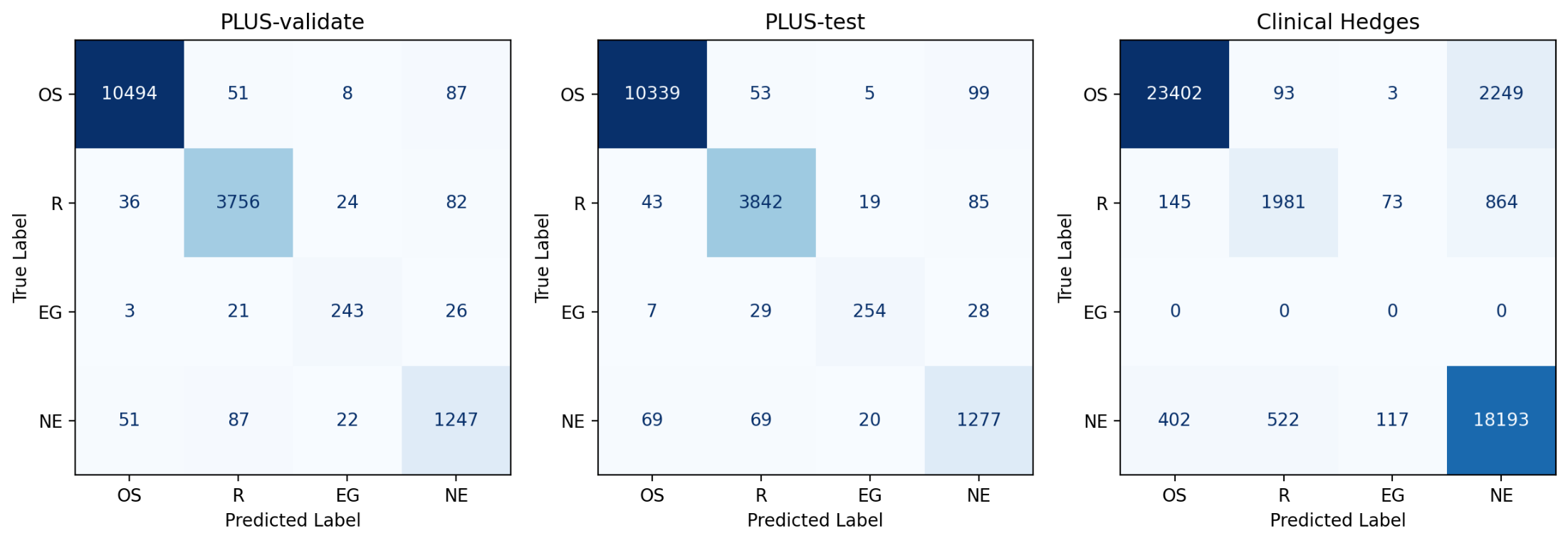


**EG** Evidence-based guideline; **NE** Non-experimental; **OS** Original study; **R** Review.

# **Figure S12.** Confusion matrices for the best-F2 model (BiomedBERT; CW: Yes; LR: 3E-5; BS: 128; WR: 0.05; WD: 0.015)


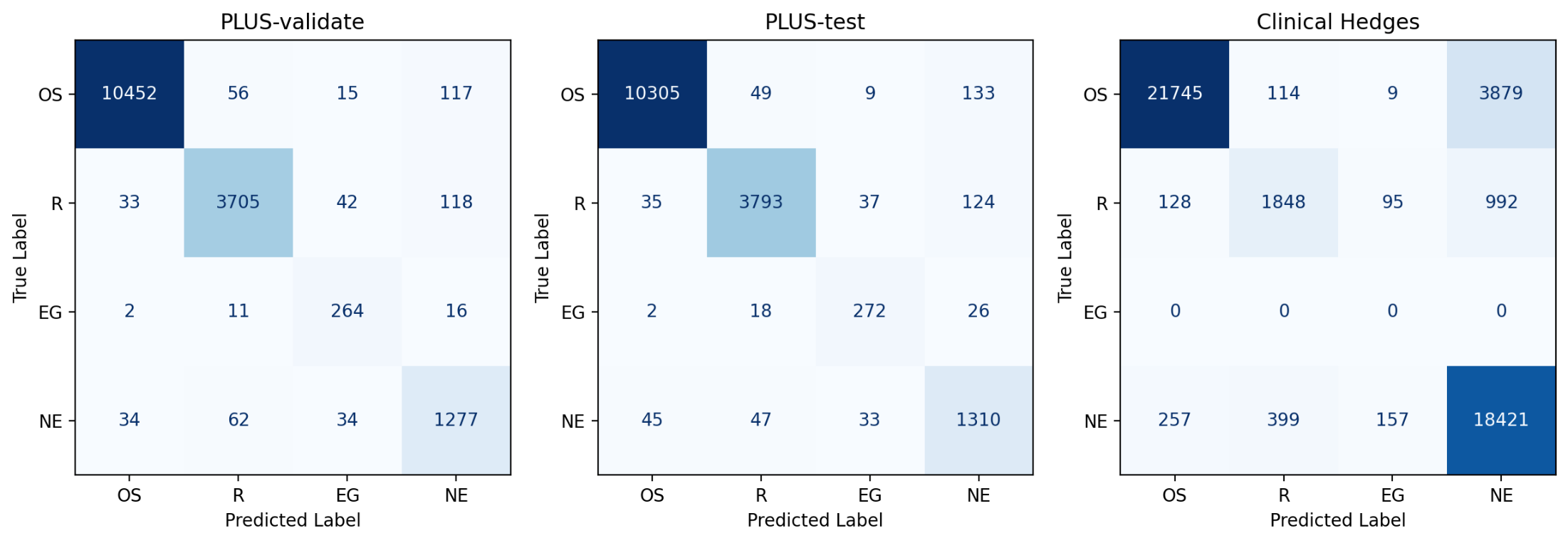


**EG** Evidence-based guideline; **NE** Non-experimental; **OS** Original study; **R** Review.

.

# **Figure S13.** Calibration plots for the best-cross entropy loss model (BioBERT; CW: No; LR: 5E-5; BS: 256; WR: 0.10; WD: 0.015)


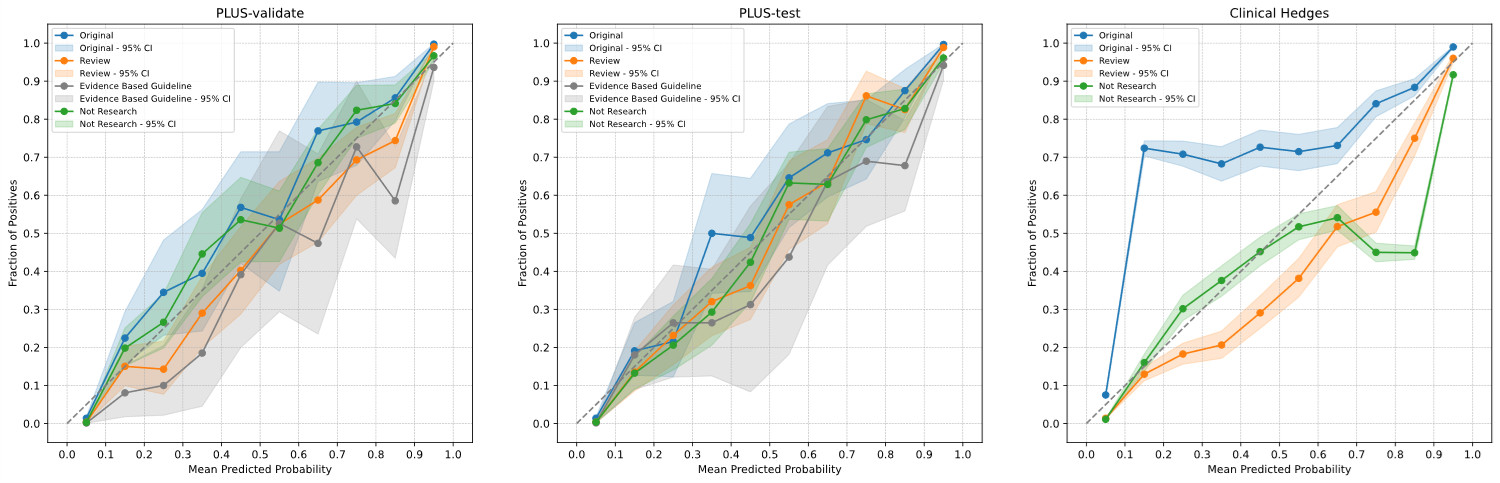


# **Figure S14.** Calibration plots for the best-Brier score model (BioBERT; CW: No; LR: 1E-5; BS: 64; WR: 0.20; WD: 0.015)

**
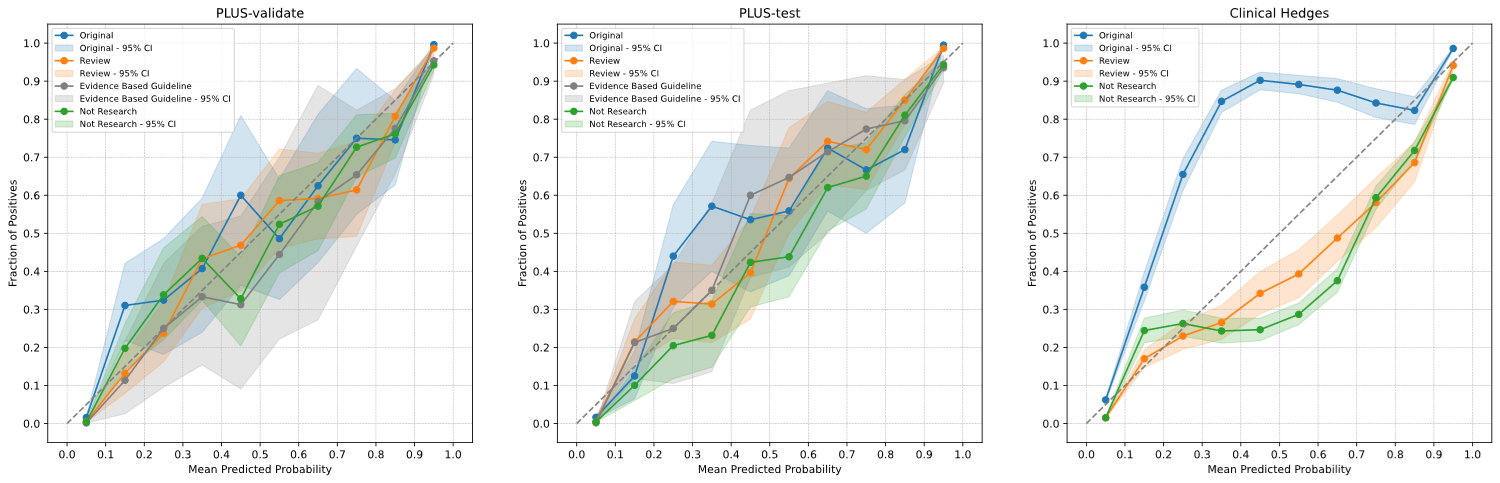
**

# **Figure S15.** Calibration plots for the best-AP model (BiomedBERT; CW: No; LR: 1E-5; BS: 128; WR: 0.05; WD: 0.010)

**
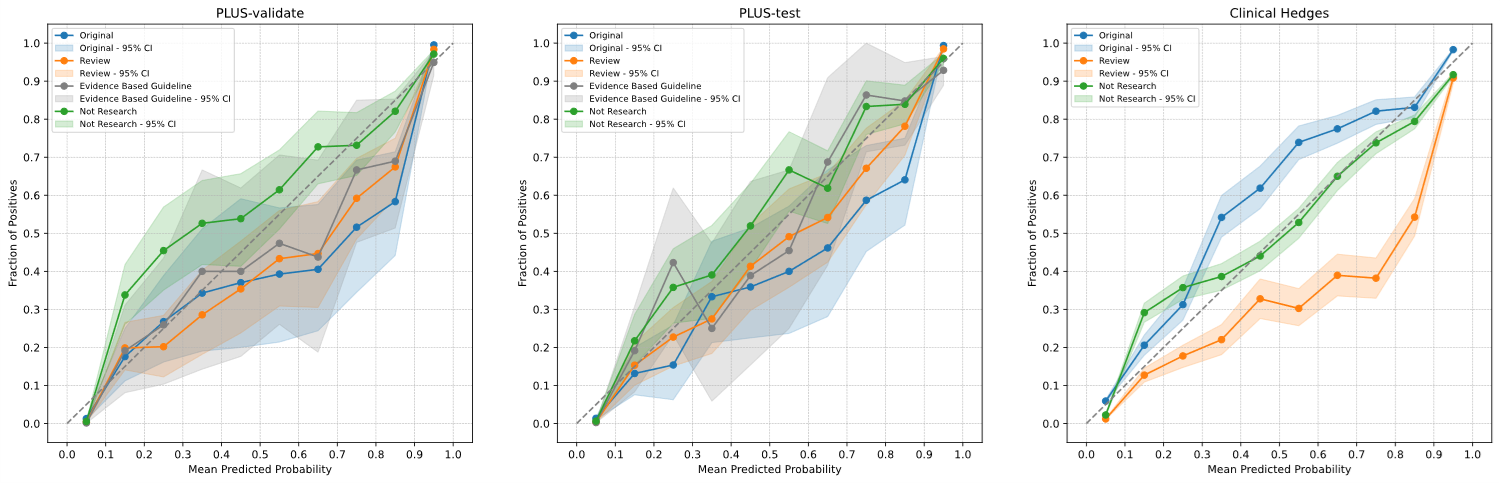
**

# **Figure S16.** Calibration plots for the best-recall model (SciBERT-uncased; CW: Yes; LR: 3E-5; BS: 256; WR: 0.05; WD: 0.010)

**
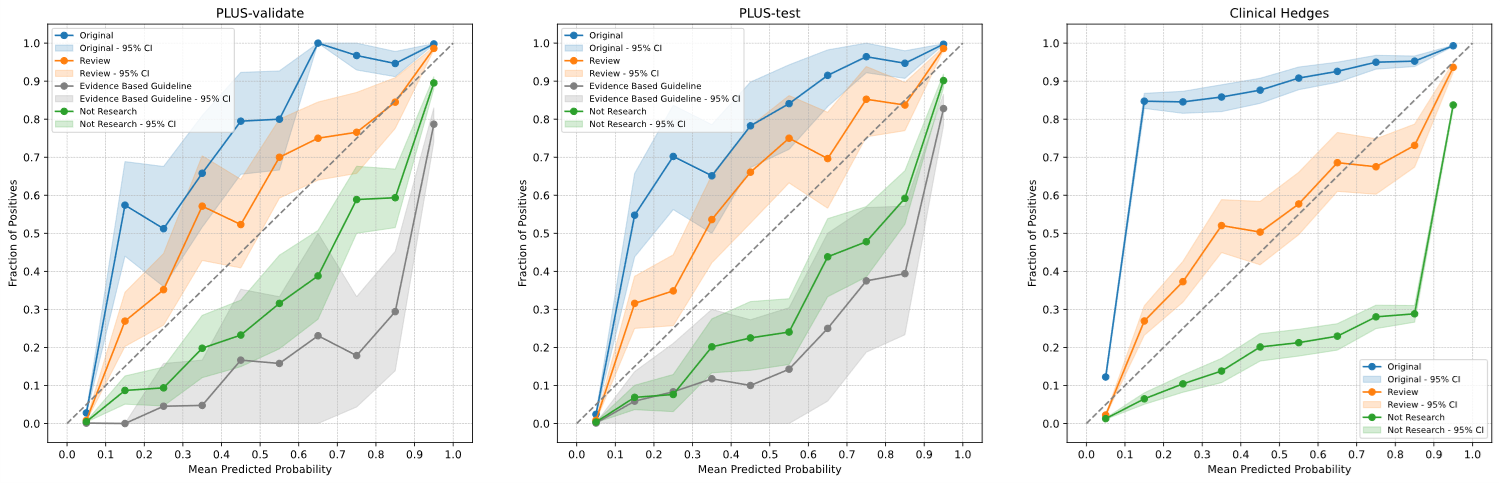
**

# **Figure S17.** Calibration plots for the best-precision model (BioLinkBERT; CW: No; LR: 3E-5; BS: 16; WR: 0.10; WD: 0.010)

**
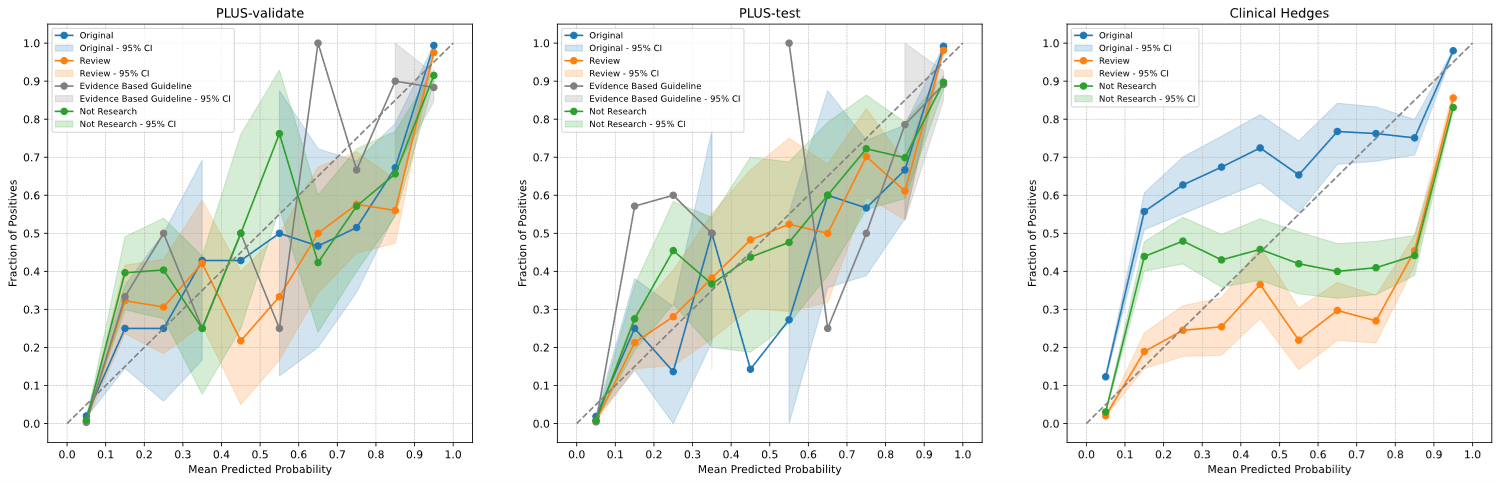
**

# **Figure S18.** Calibration plots for the best-accuracy model (BioBERT; CW: No; LR: 1E-5; BS: 256; WR: 0.05; WD: 0.015)


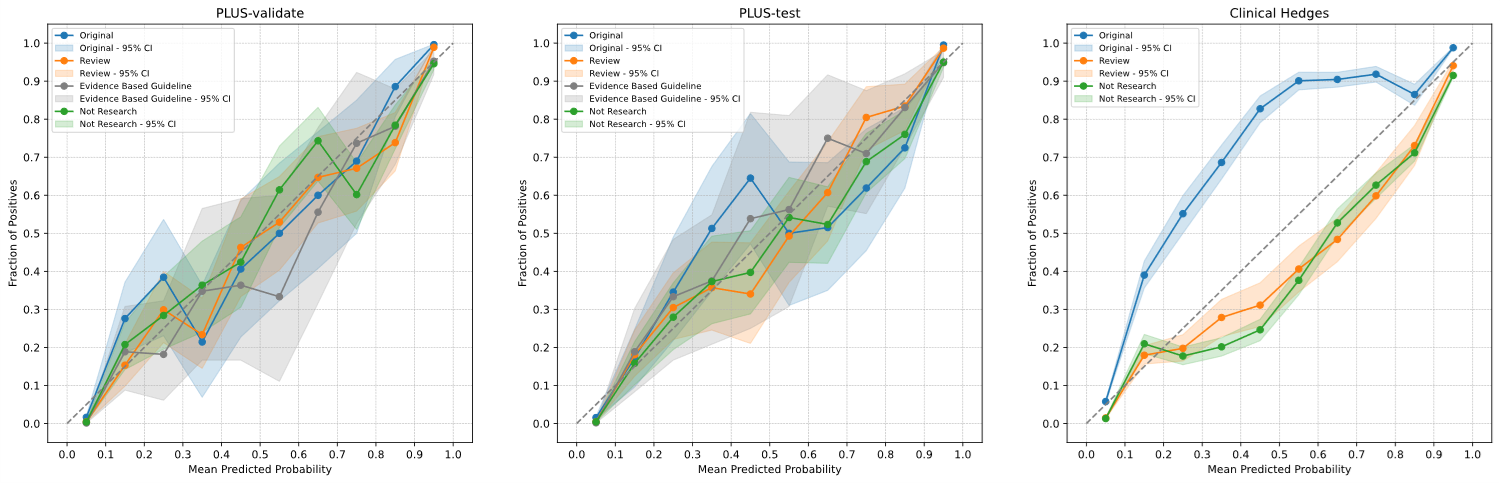


# **Figure S19.** Calibration plots for the best-F2 model (BiomedBERT; CW: Yes; LR: 3E-5; BS: 128; WR: 0.05; WD: 0.015)


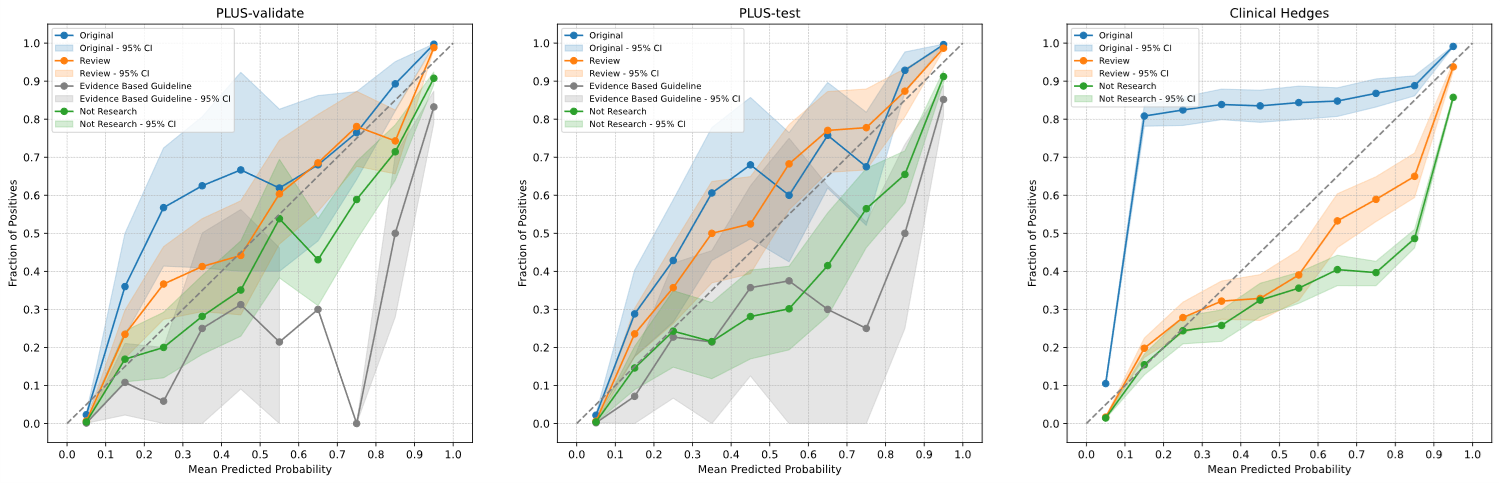

Supplement: Multimedia Appendix 1 [file ai_v5i1e77311_app1.doc]
