# Supplementary material for: Fine-Tuning and Benchmarking Transformer Models for Multiclass Classification of Clinical Research Papers: Retrospective Modeling Study
Source: JMIR AI. 2026 Apr 29;5:e77311. doi: 10.2196/77311 (PMC13173073; doi:10.2196/77311)
Supplement: Multimedia Appendix 2 [file ai_v5i1e77311_app2.docx]

**Table S1.** Macro averages of evaluation metrics on the PLUS^a^-validate set by model configuration parameters.

| Configuration and models | | Cross-entropy loss, mean (95% CI) | Brier score, mean (95% CI) | AP^b^, mean (95% CI) | Recall, mean (95% CI) | Precision, mean (95% CI) | Accuracy, mean (95% CI) | *F*_2_-score, mean (95% CI) |
| --- | --- | --- | --- | --- | --- | --- | --- | --- |
| **Pretrained model** | | | | | | | | |
|  | BioBERT^c^ | 0.055 (0.054-0.056) | 0.014 (0.014-0.014) | 0.948 (0.947-0.949) | 0.923 (0.921-0.924) | 0.877 (0.875-0.880) | 0.982 (0.982-0.982) | 0.911 (0.910-0.912) |
|  | BioELECTRA^d^ | 0.056 (0.055-0.057) | 0.014 (0.014-0.014) | 0.945 (0.943-0.946) | 0.917 (0.915-0.918) | 0.881 (0.878-0.883) | 0.982 (0.982-0.982) | 0.907 (0.907-0.908) |
|  | BioLinkBERT^e^ | 0.055 (0.054-0.056) | 0.014 (0.014-0.014) | 0.948 (0.947-0.949) | 0.921 (0.920-0.923) | 0.880 (0.877-0.882) | 0.982 (0.982-0.982) | 0.911 (0.910-0.912) |
|  | BiomedBERT^f^ (abstract only) | 0.059 (0.056-0.062) | 0.015 (0.014-0.016) | 0.943 (0.939-0.947) | 0.917 (0.913-0.921) | 0.878 (0.874-0.883) | 0.981 (0.979-0.983) | 0.907 (0.903-0.912) |
|  | BiomedBERT (abstract+full text) | 0.056 (0.055-0.057) | 0.014 (0.014-0.014) | 0.947 (0.946-0.948) | 0.920 (0.919-0.921) | 0.879 (0.877-0.882) | 0.982 (0.982-0.982) | 0.910 (0.909-0.910) |
|  | SciBERT^g^-cased | 0.057 (0.056-0.058) | 0.015 (0.014-0.015) | 0.946 (0.945-0.947) | 0.918 (0.917-0.920) | 0.876 (0.873-0.879) | 0.982 (0.981-0.982) | 0.908 (0.907-0.909) |
|  | SciBERT-uncased | 0.056 (0.055-0.057) | 0.014 (0.014-0.015) | 0.946 (0.945-0.947) | 0.919 (0.917-0.921) | 0.880 (0.878-0.883) | 0.982 (0.982-0.982) | 0.909 (0.908-0.910) |
| **CW^h^ adjustment** | | | | | | | | |
|  | No | 0.049 (0.049-0.050) | 0.013 (0.013-0.013) | 0.951 (0.950-0.951) | 0.912 (0.911-0.912) | 0.897 (0.896-0.897) | 0.983 (0.983-0.983) | 0.908 (0.908-0.909) |
|  | Yes | 0.063 (0.062-0.064) | 0.016 (0.015-0.016) | 0.942 (0.940-0.943) | 0.927 (0.926-0.928) | 0.861 (0.860-0.863) | 0.981 (0.980-0.981) | 0.910 (0.909-0.911) |
| **LR^i^** | | | | | | | | |
|  | 1 × 10^–5^ | 0.054 (0.053-0.054) | 0.014 (0.014-0.014) | 0.951 (0.951-0.952) | 0.921 (0.920-0.922) | 0.886 (0.884-0.887) | 0.983 (0.983-0.983) | 0.912 (0.912-0.912) |
|  | 3 × 10^–5^ | 0.057 (0.055-0.058) | 0.015 (0.014-0.015) | 0.946 (0.944-0.947) | 0.919 (0.918-0.921) | 0.878 (0.875-0.880) | 0.982 (0.981-0.982) | 0.909 (0.907-0.911) |
|  | 5 × 10^–5^ | 0.059 (0.058-0.059) | 0.015 (0.015-0.015) | 0.942 (0.940-0.943) | 0.918 (0.917-0.919) | 0.873 (0.871-0.875) | 0.981 (0.981-0.981) | 0.906 (0.906-0.907) |
| **BS^j^** | | | | | | | | |
|  | 16 | 0.063 (0.062-0.063) | 0.015 (0.015-0.015) | 0.937 (0.936-0.939) | 0.911 (0.910-0.912) | 0.883 (0.881-0.884) | 0.982 (0.982-0.982) | 0.904 (0.904-0.905) |
|  | 32 | 0.057 (0.056-0.058) | 0.014 (0.014-0.015) | 0.944 (0.943-0.945) | 0.918 (0.917-0.919) | 0.880 (0.878-0.882) | 0.982 (0.982-0.982) | 0.909 (0.908-0.909) |
|  | 64 | 0.056 (0.055-0.056) | 0.014 (0.014-0.014) | 0.948 (0.948-0.949) | 0.921 (0.919-0.922) | 0.878 (0.876-0.881) | 0.982 (0.982-0.982) | 0.910 (0.909-0.910) |
|  | 128 | 0.053 (0.052-0.054) | 0.014 (0.014-0.014) | 0.951 (0.950-0.951) | 0.923 (0.922-0.925) | 0.877 (0.875-0.879) | 0.982 (0.982-0.982) | 0.912 (0.911-0.912) |
|  | 256 | 0.054 (0.051-0.056) | 0.014 (0.013-0.015) | 0.950 (0.948-0.953) | 0.923 (0.920-0.926) | 0.876 (0.872-0.880) | 0.981 (0.980-0.983) | 0.911 (0.908-0.914) |
| **WR^k^** | | | | | | | | |
|  | 0.05 | 0.057 (0.056-0.057) | 0.014 (0.014-0.015) | 0.946 (0.945-0.947) | 0.921 (0.920-0.921) | 0.877 (0.875-0.878) | 0.982 (0.982-0.982) | 0.910 (0.909-0.910) |
|  | 0.10 | 0.056 (0.055-0.057) | 0.014 (0.014-0.014) | 0.946 (0.946-0.947) | 0.920 (0.919-0.921) | 0.878 (0.877-0.880) | 0.982 (0.982-0.982) | 0.909 (0.908-0.910) |
|  | 0.20 | 0.056 (0.055-0.058) | 0.014 (0.014-0.015) | 0.946 (0.944-0.948) | 0.918 (0.916-0.920) | 0.881 (0.879-0.884) | 0.982 (0.981-0.983) | 0.909 (0.907-0.910) |
| **WD^l^** | | | | | | | | |
|  | 0.005 | 0.056 (0.055-0.057) | 0.014 (0.014-0.014) | 0.946 (0.946-0.947) | 0.920 (0.919-0.921) | 0.879 (0.877-0.881) | 0.982 (0.982-0.982) | 0.909 (0.909-0.910) |
|  | 0.010 | 0.056 (0.056-0.057) | 0.014 (0.014-0.014) | 0.946 (0.945-0.947) | 0.920 (0.919-0.920) | 0.879 (0.877-0.881) | 0.982 (0.982-0.982) | 0.909 (0.909-0.910) |
|  | 0.015 | 0.057 (0.055-0.058) | 0.014 (0.014-0.015) | 0.946 (0.944-0.948) | 0.919 (0.917-0.921) | 0.879 (0.876-0.881) | 0.982 (0.981-0.982) | 0.909 (0.907-0.910) |

^a^PLUS: Premium Literature Service.

^b^AP: average precision.

^c^BioBERT: Biomedical Bidirectional Encoder Representations from Transformers (fine-tuned on biomedical text).

^d^BioELECTRA: Biomedical Efficiently Learning an Encoder that Classifies Token Replacements Accurately.

^e^BioLinkBERT: Biomedical Document Link Bidirectional Encoder Representations from Transformers.

^f^BiomedBERT: Biomedical Bidirectional Encoder Representations from Transformers (trained entirely on biomedical text). Formerly known as PubMedBERT.

^g^SciBERT: Scientific Bidirectional Encoder Representations from Transformers.

^h^CW: class weight.

^i^LR: learning rate.

^j^BS: batch size.

^k^WR: warmup ratio.

^l^WD: weight decay.

**Table S2.** Performance of the top models on AUROC^a^, *F*_1_-score, and MCC^b^.

| Model (best metric; CW^c^, LR^d^, BS^e^, WR^f^, WD^g^) and class | | PLUS^h^-validate | | |
| --- | --- | --- | --- | --- |
|  |  | AUROC, score (bootstrapped 95% CI) | *F*_1_-score, score (bootstrapped 95% CI) | MCC, score (bootstrapped 95% CI) |
|  | |  |  |  |
| **BiomedBERT^i^ (AUROC; no, 3 × 10^–5^, 256, 0.20, 0.005)** | | | | |
|  | Original study | 0.998 (0.997-0.998) | 0.988 (0.987-0.990) | 0.966 (0.962-0.971) |
|  | Review | 0.997 (0.997-0.998) | 0.959 (0.955-0.964) | 0.947 (0.940-0.953) |
|  | Evidence-based guideline | 0.997 (0.996-0.998) | 0.837 (0.804-0.868) | 0.834 (0.800-0.866) |
|  | Nonexperimental paper | 0.992 (0.990-0.994) | 0.867 (0.854-0.880) | 0.855 (0.840-0.869) |
|  | Macro average | 0.996 (0.995-0.997) | 0.913 (0.903-0.923) | 0.901 (0.890-0.911) |
| **BioBERT^j^ (*F*_1_-score; no, 5 × 10^–5^, 64, 0.10, 0.015)** | | | | |
|  | Original study | 0.997 (0.997-0.998) | 0.988 (0.987-0.990) | 0.967 (0.963-0.971) |
|  | Review | 0.997 (0.997-0.998) | 0.960 (0.956-0.964) | 0.947 (0.942-0.953) |
|  | Evidence-based guideline | 0.996 (0.993-0.997) | 0.836 (0.801-0.867) | 0.833 (0.798-0.864) |
|  | Nonexperimental paper | 0.991 (0.989-0.993) | 0.873 (0.860-0.885) | 0.861 (0.847-0.873) |
|  | Macro average | 0.995 (0.994-0.996) | 0.914 (0.904-0.924) | 0.902 (0.892-0.912) |
| **BiomedBERT (MCC; no, 1 × 10^–5^, 16, 0.20, 0.005)** | | | | |
|  | Original study | 0.997 (0.997-0.998) | 0.989 (0.987-0.990) | 0.967 (0.963-0.972) |
|  | Review | 0.997 (0.996-0.998) | 0.961 (0.956-0.965) | 0.949 (0.943-0.954) |
|  | Evidence-based guideline | 0.994 (0.991-0.997) | 0.837 (0.805-0.866) | 0.835 (0.802-0.864) |
|  | Nonexperimental paper | 0.991 (0.989-0.993) | 0.870 (0.856-0.883) | 0.857 (0.843-0.872) |
|  | Macro average | 0.995 (0.994-0.996) | 0.914 (0.904-0.923) | 0.902 (0.892-0.912) |

^a^AUROC: area under the receiver operating characteristic curve.

^b^MCC: Matthew’s correlation coefficient.

^c^CW: class weight.

^d^LR: learning rate.

^e^BS: batch size.

^f^WR: warmup ratio.

^g^WD: weight decay.

^h^PLUS: Premium Literature Service.

^i^BiomedBERT: Biomedical Bidirectional Encoder Representations from Transformers (trained entirely on biomedical text). Formerly known as PubMedBERT.

^j^BioBERT: Biomedical Bidirectional Encoder Representations from Transformers (fine-tuned on biomedical text).
